# Recombination Mediator Proteins: Misnomers That Are Key to Understanding the Genomic Instabilities in Cancer

**DOI:** 10.3390/genes13030437

**Published:** 2022-02-27

**Authors:** Justin Courcelle, Travis K. Worley, Charmain T. Courcelle

**Affiliations:** Department of Biology, Portland State University, Portland, OR 97201, USA; tworley@pdx.edu (T.K.W.); charmc@pdx.edu (C.T.C.)

**Keywords:** RecF, RecO, RecR, RecJ, RecQ, nucleotide excision repair, translesion synthesis, recombination

## Abstract

Recombination mediator proteins have come into focus as promising targets for cancer therapy, with synthetic lethal approaches now clinically validated by the efficacy of PARP inhibitors in treating BRCA2 cancers and RECQ inhibitors in treating cancers with microsatellite instabilities. Thus, understanding the cellular role of recombination mediators is critically important, both to improve current therapies and develop new ones that target these pathways. Our mechanistic understanding of BRCA2 and RECQ began in *Escherichia coli*. Here, we review the cellular roles of RecF and RecQ, often considered functional homologs of these proteins in bacteria. Although these proteins were originally isolated as genes that were required during replication in sexual cell cycles that produce recombinant products, we now know that their function is similarly required during replication in asexual or mitotic-like cell cycles, where recombination is detrimental and generally not observed. Cells mutated in these gene products are unable to protect and process replication forks blocked at DNA damage, resulting in high rates of cell lethality and recombination events that compromise genome integrity during replication.

## Contents

1. Nonrecombinational roles of recombination mediators during replication
2. Replication substrates created during encounters with DNA damage
3. The RecF pathway maintains replication following disruption by DNA damage
4. RecF, RecO, and RecR are required to maintain the replication fork and restore processive replication
5. RecJ and RecQ process the replication forks following DNA damage to promote resumption from the site where disruption occurred
6. Evidence that lesion repair occurs at disrupted replication forks to prevent mutations or rearrangements by translesion synthesis or recombination
7. Maintaining and restoring processive replication is specific to the RecF pathway
8. Recombination mediators are functionally conserved between bacteria and mammals

## 1. Nonrecombinational Roles of Recombination Mediators during Replication

The RecF pathway gene products are often referred to as recombinational mediators and include RecF, RecO, and RecR, along with the processing enzymes RecJ and RecQ. The manner in which a gene is discovered can have a profound effect upon, and often bias, the way in which we think about its cellular function. Meiotic cell cycles, despite being extremely rare, have received a disproportionate amount of scientific attention relative to their mitotic counterparts. Unlike mitosis, these rare specialized cell cycles scramble the genome rather than maintain it, producing ‘hybrid’ or recombinant DNA molecules derived from two different parents. Although bacteria were long considered to be asexual, following the discovery of conjugation and natural transformation, it became clear that bacteria also had true sexual reproductive cycles that produced hybrid DNA molecules [1]. Recognizing the parallel between bacterial sex and meiosis, Clark and Margulies undertook a genetic screen with the goal of identifying the ‘rec’ enzymes that were required to produce the recombination events (rearrangements) generating genetic diversity during sexual cycles using *E. coli* as a model [2,3,4]. It quickly became apparent that mutations in many of these gene products also rendered cells hypersensitive to DNA damage during normal growth [2,5], indicating that these proteins functioned during asexual cell cycles as well. At the time, the only known property of these proteins was that they were required for recombinational products to form during sex. As such, models were developed that speculated recombination occurred in asexual cells as a mechanism of repair [6,7].

**Figure 1:**
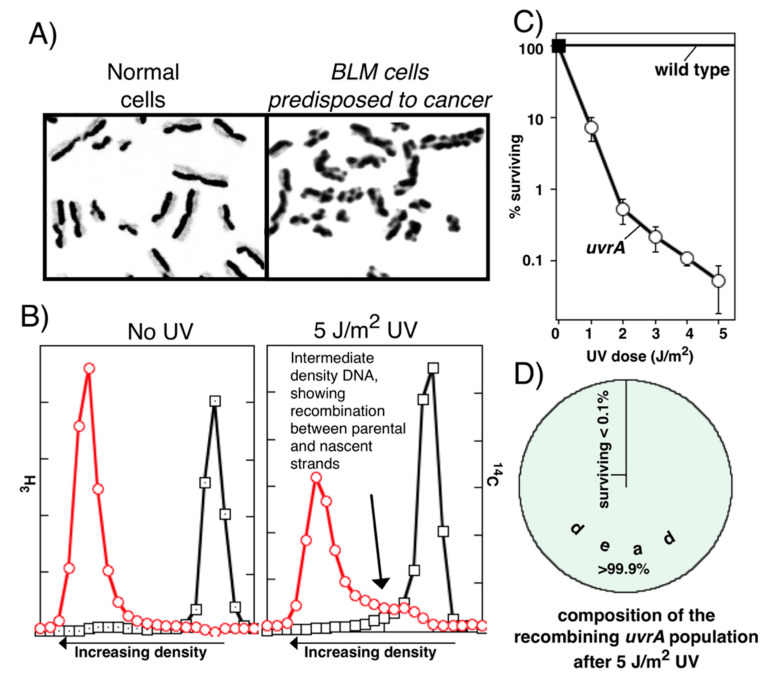
Recombination frequency during nonsexual cell cycles correlates with genomic instability and cell death. (**A**) One form of recombination, sister chromatid exchanges, can be observed directly by growing cultured cells in 5-bromo-deoxyuridine for two generations before staining with Giemsa. In healthy cells, few exchanges are observed. By contrast, in cells lacking BLM, a RecQ homolog, recombination events are observed more frequently, and patients are predisposed to developing cancer. RecQ in *E. coli* is similarly required to suppress illegitimate recombination from occurring [17]. Photos generously provided by S. Wolff and A. A. Sandberg. (**B**) Experiment demonstrating that DNA damage-induced recombinational exchanges can be observed during replication in *E. coli*. By growing cells in different isotopic media, the DNA made before and after UV irradiation can be separated based upon their buoyant density in isopycnic alkali CsCl gradients. To test whether recombination can be induced by UV lesions, *uvrA* mutants defective in nucleotide excision repair were UV irradiated and allowed to recover for one hour. Unlike unirradiated *uvrA* mutants or irradiated wild-type cells, the DNA made in irradiated *uvrA* cultures contained more DNA at an intermediate density, demonstrating exchanges between the parental and daughter DNA strands are occurring in these cells (redrawn from [18] which reproduce results reported in [7]). (**C**) and (**D**) Although these exchanges were originally interpreted to represent a repair mechanism, the populations in which they were observed and characterized were rendered inviable by the UV treatment.

However, this early proposal set up a paradox that is difficult to resolve with what is observed in humans and the development of cancer. Essentially, it implies that since recombination is acting as a repair mechanism, the more recombination events observed after DNA damage, the better off the cells should be. However, unlike the specialized events occurring in sex cells, strand exchanges are not normally observed during asexual replicative cycles. Additionally, when recombination events are observed, they are detrimental to the genomic stability and viability of the cell (Figure 1A). In the clinic, mitotic strand exchange frequencies are used diagnostically and prognostically as markers for cancer and correlate directly with genomic instability, cell death, and carcinogenic transformation [8,9,10,11,12,13,14,15,16].

The same correlation between cell death, genome instability, and the frequency of recombination also occurs in bacteria. In populations undergoing extensive recombination, high levels of genomic instability and cell death also occur [6,7,19,20,21,22,23,24,25,26,27,28,29,30,31,32,33,34,35,36,37,38,39,40,41,42,43,44,45,46,47,48]. This concept is often lost because in the initial studies that characterized replication following DNA damage, recombination was hypothesized to function as a repair mechanism (Figure 1B). However, in order to observe strand exchanges after DNA damage, these initial studies found it necessary to carry out their experiments in repair-deficient strains (*uvr* mutants), using doses that were lethal for the populations in which the exchanges were characterized [6,7,49] (Figure 1C,D). Essentially all subsequent studies followed this example [6,7,19,20,21,22,23,24,25,26,27,28,29,30,31,32,33,34,35,36,37,38,39,40,41,42,43,44,45,46,47,48], excluding DNA repair as a step in the recovery process, and characterizing the process in *uvr* mutant populations where few of the cells remained viable. Thus, one cannot separate the presence of recombination events from cell death and genomic instability. Additionally, although it was not considered in these original articles, the lack of viability in cells where the recombination events are characterized makes a strong argument for the idea that DNA damage-induced recombination is not acting as a mechanism of repair, but is an aberrant, sometimes lethal, event that results when the normal pathway of recovery is impaired or cannot occur.

We now know that the RecF pathway proteins function to maintain semiconservative replication, both during sexual cycles that produce recombinant progeny, and asexual cycles that produce clonal progeny [17,18,50,51,52,53,54,55,56,57,58,59,60,61,62,63,64]. Several RecF pathway genes are intimately associated with the process of DNA replication, even at the level of genomic organization. *recF* is located in the same operon as *gyrB*, a subunit of the replicative gyrase, and *dnaN*, the processivity subunit of the replisome [65,66]. *recR* is found in the same operon as *dnaXZ*, encoding the tau and gamma subunits of the replisome [66,67]. *recO* is found in the same operon as *era*, a gene encoding an essential G protein [68], which can suppress primase defects when mutated [69,70,71].

## 2. Replication Substrates Created during Encounters with DNA Damage

**Figure 2:**
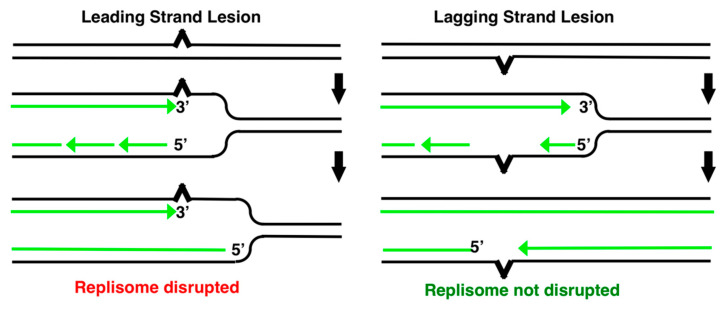
Substrates generated during replication on UV-damaged templates. Based upon our current understanding, lesions in the leading-strand template disrupt replisome progression. In contrast, lesions in the lagging-strand template produce gaps but do not disrupt replication.

One interesting feature of the substrates generated by replicational encounters with DNA damage is that their structure varies depending upon which template strand contains the DNA lesion. Since DNA polymerization on both strands occurs in a 5′–3′ direction, coordinated replication of both templates requires unique enzymatic dynamics for each strand. While the leading-strand template can be synthesized in a continuous, processive 5′–3′ manner, the lagging-strand template must be synthesized in a direction opposite to the fork’s progress, and requires a primase activity that constantly “reinitiates” the lagging strand polymerization, resulting in discontinuous synthesis of the template (Okazaki fragments). These alternative mechanisms of synthesis present different problems for the replication machinery when it encounters a DNA lesion. In reconstituted systems and in vivo on plasmid substrates, several labs have shown that a blocking lesion in the leading-strand template disrupts the replisome machinery, arresting replisome progression [72,73,74,75,76]. In contrast, lesions in the lagging-strand template do not disrupt the progression of the replisome, but result in the production of a gap on opposite the strand containing the lesion (Figure 2). On the chromosome, the transient disruption of replication by UV is associated with an overall inhibition and the production of some limited gapped fragments, consistent with the products observed in vitro [6,18,21,55,77]. In this review, we focus primarily on those events and observations associated with lesions that disrupt the replisome’s progression and the mechanism that restores it.

## 3. The RecF Pathway Maintains Replication Following Disruption by DNA Damage

**Figure 3:**
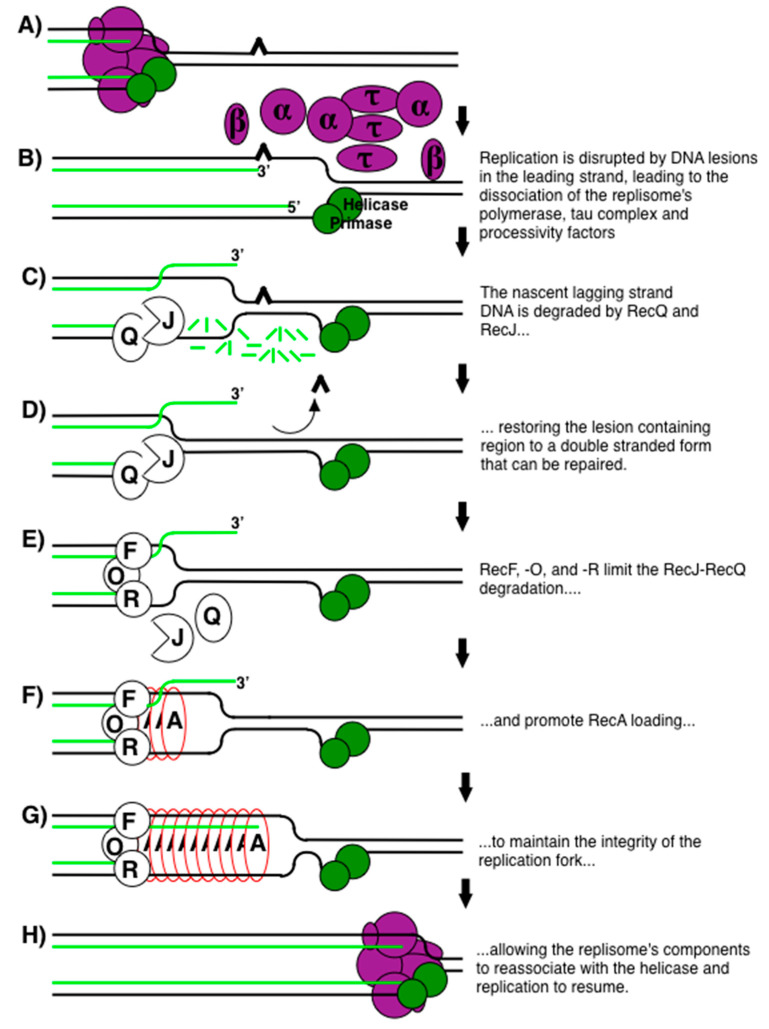
Model of how the RecF pathway restores replication following disruption. ^, pyrimidine dimer; purple subunits, the tau complex, DNA polymerase III, and processivity factors of the replisome; green subunits, helicase-primase; Q, RecQ; J, RecJ: F, RecF; O, RecO; R, RecR; A, RecA. (**A**–**H**) Different RecF pathway gene products.

The current model for how RecF pathway gene products maintain replication and suppress illegitimate recombination is shown in Figure 3, and the supporting experimental evidence from studies using UV-induced DNA damage as a model lesion are presented in the sections that follow. 254-nm UV light induces cyclobutane pyrimidine dimers and 6-4 photoproducts that block DNA polymerases and disrupt replisome progression when encountered in the leading strand (Figure 3A) [50,72,73,77,78]. We use the term disruption to distinguish it from replisome pausing or inactivation. These latter terms might imply that the replisome could simply resume once a missing precursor or cofactor is resupplied. In *E. coli*, the disruption of replication at UV-induced lesions involves the uncoupling of leading and lagging strand synthesis, exposure of the nascent lagging-strand DNA to exonucleolytic degradation, and the partial dissociation of the replisome’s components [50,51,53,56,58,73,74]. The DNA polymerase, beta clamp, and tau subunits of the replisome dissociate from the arrested replication fork (Figure 3B) [58]. Dissociation of these subunits, even in the absence of DNA damage, is sufficient to induce processing events that mimic those seen after DNA damage is encountered [58]. In contrast, both helicase and primase remain associated with the branched replication fork DNA [58,74,79]. The integrity of the replication fork DNA structure remains intact throughout the recovery process, as no ‘collapsed forks’, double-strand breaks, or broken linear DNA are observed [53,57,80].

The partial disruption of the replisome exposes the nascent DNA to enzymatic degradation by RecJ, a 5′–3′ single-strand exonuclease, and RecQ, a complementary 3′–5′ helicase (Figure 3C) [51,53,81,82]. Together, these enzymes partially degrade the nascent lagging strand of the replication fork, which restores the lesion-containing region to a double-stranded form that can be repaired by nucleotide excision repair (Figure 3D) [18,51,52,53,56,83,84]. The leading strand appears to remain protected, as degradation on this strand is not detected [51,57,79]. Presumably, some partial displacement of the leading strand must also occur. We would speculate that this displacement could occur spontaneously due to positive supercoiling created in the template ahead of the replication fork [85,86]; however, it also remains possible that other, as yet unidentified, enzymes may catalyze this reaction. Some have postulated that branch migration enzymes, such as RecG or RuvAB, may catalyze this step [87,88,89,90]. However, these enzymes do not appear to be required for this reaction in vivo since replication fork processing and resumption of DNA synthesis occur normally in mutants lacking these enzymes [80,91]. In the absence of RecJ or RecQ, repair cannot occur and the recovery is delayed for 50 min, a time corresponding to when UmuDC translesion polymerase is activated [56]. In *recJ* and *recQ* mutants, both Cell survival and the resumption of replication become entirely dependent on translesion DNA synthesis [56]. Additionally, this processing plays a key role in suppressing illegitimate recombination, by creating a large single-stranded region on the lagging-strand template at the replication fork for RecA to bind and pair with its sister chromatid [92,93,94,95,96]. This protects the DNA from degradation and helps to ensure that the 3′-end of the disrupted replication fork resumes on the same template, at the same site, from where disruption occurred [17,51,62,63,64,97].

RecF, RecO, and RecR function to maintain the integrity of the replication fork DNA and are required for the replisome to reassemble and resume [50,53,54,58,79]. Together, these proteins limit the degradation by RecJ and RecQ, facilitate RecA loading onto the disrupted replisome substrate, which protects the DNA from degradation, and re-pairs the 3′ nascent leading strand with its template (Figure 3E–G). Resumption likely requires RecA to re-pair these strands to create a substrate that the replisome could then resume from. Collectively, RecF pathway activities may provide the substrate necessary for DNA polymerase, and other replisome subunits to reassociate with the helicase-primase complex and restore synthesis.

The ability to resume DNA synthesis depends heavily on the removal of the UV lesions by the UvrABC excinuclease complex which incises a 12-bp region surrounding the damaged bases [18,50,77,98,99]. UvrD helicase, DNA polymerase I, and ligase then remove, resynthesize, and join the missing bases to complete repair [100,101,102]. In *uvrA*, *uvrB*, or *uvrC* mutants, these lesions are not removed, the replication forks remain blocked, and the resumption of replication is severely impaired [18,49,53,77,103]. In these repair-defective populations, high frequencies of chromosomal exchanges and extensive cell death occur [5,6,7,18,52,53,83,84]. In repair-proficient cells, these recombination events are efficiently suppressed, survival is greatly enhanced, and a robust recovery of replication is observed, indicating that the normal mechanism of replication recovery is integrated with lesion repair [7,18,49,52,77,83,84].

## 4. RecF, RecO, and RecR Are Required to Maintain the Replication Fork and Restore Processive Replication

**Figure 4:**
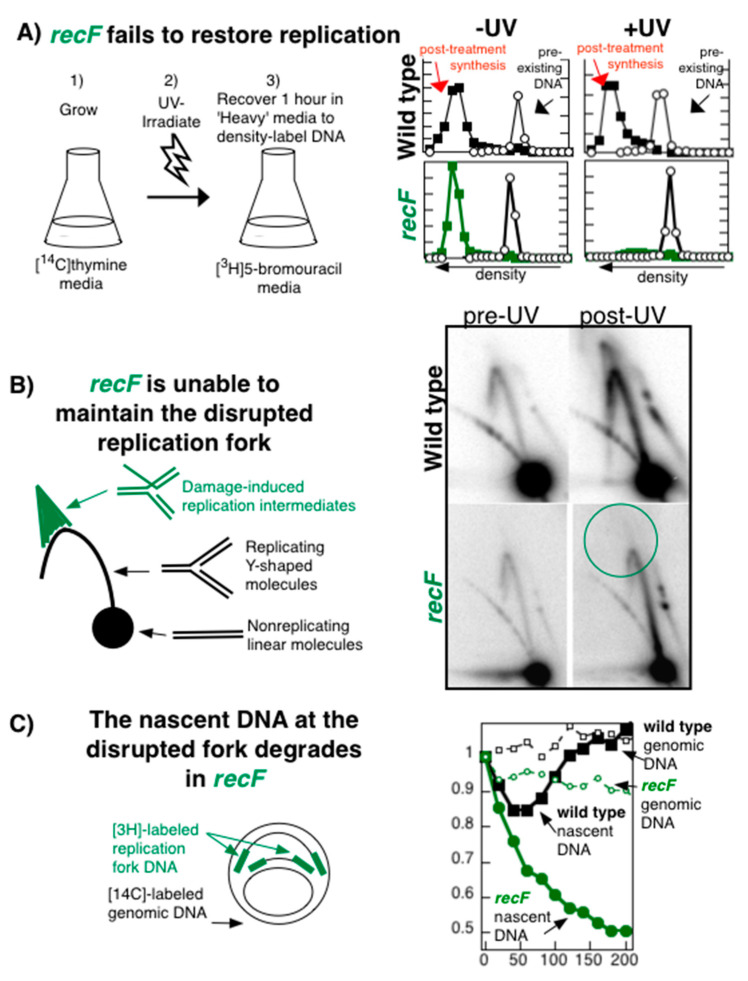
RecF is required to maintain replication forks disrupted by DNA damage. (**A**) *recF mutants fail to restore replication following disruption by UV-induced damage*. The amount of replication occurring within 1 h after UV irradiation with 25 J/m^2^ was analyzed by alkaline CsCl density gradients. Cells prelabeled with [^14^C]thymine were irradiated or mock-irradiated, filtered, and allowed to recover for 1 h in media containing [^3^H]5-bromodeoxyuridine to density label DNA made during this period. Circles, ^14^C prelabeled DNA; squares, ^3^H replicated DNA. (**B**) *recF mutants are unable to maintain the replication fork processing observed by two-dimensional gel electrophoresis*. The migration pattern in 2D agarose gels for linearized pBR322 plasmid after UV treatment. Nonreplicating plasmids run as a linear 4.4 kb fragment. Normal replicating plasmids form Y-shaped structures and migrate more slowly due to their increased size and nonlinear shape, moving as an arc that extends from the linear fragment. Replication intermediates observed after UV damage form double-Y or X-shaped structures that migrate in the cone region. Whereas processing intermediates transiently accumulate in wild-type cells after UV irradiation, no intermediates are observed in *recF* mutants. (**C**) *The nascent DNA undergoes excessive degradation in recF mutants*. [^3^H]thymidine is added to [^14^C]thymine-prelabeled cells for 10 s immediately before the cells were filtered and irradiated with 25 J/m^2^ in nonradioactive medium. The fraction of the radioactivity remaining in the DNA is plotted against time. Loss of ^14^C genomic DNA (open symbols) can be compared to the loss of the ^3^H DNA synthesized at the growing fork just prior to irradiation (filled symbols). Squares, wild type; circles, *recF*. Compiled and redrawn from data reported in [18,53].

If the action of a gene product is required to restore replication forks that are blocked by DNA damage, then one would predict that replication would fail to resume in its corresponding irradiated mutant. This is the phenotype exhibited by *recF*, *recO*, or *recR* mutants. The ability to resume replication can be monitored directly using several different approaches [77,104,105,106,107]. One assay used to measure the ability to resume replication is shown in Figure 4A and involves growing the culture in 5-bromodeoxyuridine, a thymine analog, to quantify the amount of DNA that continues to accumulate after UV irradiation. Cultures receiving either 25 J/m^2^ or no irradiation are incubated in medium containing 5-bromodeoxyuridine for 1 h, so that any DNA synthesized during this period is of a greater density than that of DNA synthesized before irradiation. The denser, replicated DNA in each culture can then be separated from the rest of the DNA in isopycnic alkaline CsCl gradients and quantified. By this measure, wild-type cells have fully recovered replication within 1 h, as the amount of replicated DNA in the irradiated culture is nearly equivalent to that of the unirradiated culture. Consistent with the idea that RecF is required to restore replication forks that encounter DNA damage, no further replication is observed in mutants lacking RecF. Other approaches used to measure the resumption of replication have also confirmed these observations [50,53,91,106].

To examine the structure of the disrupted replication forks, two-dimensional (2D) agarose gel analysis has been employed to visualize the structural intermediates associated with arrested replication forks on plasmids such as pBR322 [53,105,108]. Cells containing the plasmid pBR322 are UV irradiated, and the genomic DNA is purified, digested with a restriction enzyme that linearizes the plasmid at its unidirectional origin of replication, and analyzed by 2D agarose gels at times before and after irradiation. In this technique, nonreplicating plasmids migrate as linear 4.4 kb fragments, whereas replicating fragments form Y-shaped structures and migrate more slowly because of their larger size and nonlinear shape. These replicating fragments form an arc that extends out from the linear fragment (Figure 4B). Following UV irradiation of wild-type cells, RecF, RecO, and RecR, together with RecA, are able to maintain the replication fork DNA, limiting the degradation that occurs. This produces a transient extrusion of the nascent DNA at the blocked replication fork that can be observed on 2D agarose gels [53]. The extruded nascent DNA converts the three-arm active replication fork structure into a four-arm arrested replication intermediate that further retards its mobility in the gel, migrating in a cone region beyond the normal replication arc. By contrast, in the absence of RecF, this extruded DNA is not maintained, the nascent lagging strand is extensively degraded, and the cone region intermediate is not observed.

The processing and degradation that occur at the disrupted replication fork in the cell can also be characterized by following the fate of the genomic and nascent DNA at the replication fork made just prior to irradiation [50,105]. In this approach, exponentially growing, [^14^C]thymine-prelabeled cultures are pulse labeled with [^3^H]thymidine for 10 s to label the nascent DNA at replication forks before the culture is transferred to nonradioactive medium and UV irradiated. The ^14^C prelabel allows one to compare the degradation occurring in the overall genome to that in the ^3^H-labeled DNA made at replication forks. By this technique, one observes that in UV-irradiated wild-type cells, the genomic DNA remains largely intact, while some limited degradation is detected in the nascent DNA at times prior to when replication recovers (Figure 4C) [18,50,51,53]). In principle, the precipitable ^3^H pulse label should remain constant or decrease over time in this assay. However, an increase in precipitable ^3^H label is consistently observed in samples once replication has recovered. This is likely due to remaining intracellular pools of labeled nucleotides. In contrast to the limited degradation in wild-type cells, the degradation in *recF* mutants is much more extensive, resulting in a loss of approximately half of the nascent DNA at the blocked replication forks.

Taken together, the failure to restore replication, the lack of replication processing intermediates, and the degradation of the nascent fork DNA that occurs in *recF* mutants argue that RecF is functionally required to maintain and process replication forks at UV-induced damage in cells. Both *recO* and *recR* mutants have been shown to exhibit phenotypes identical to *recF* with respect to these assays [50,53,54]. These phenotypes are likely to account for the cell death that occurs in irradiated *recF*, *recO*, and *recR* populations.

Several lines of evidence, both in vivo and in vitro, are consistent with this model and suggest that RecF, RecO, and RecR operate at a common step in loading and stabilizing RecA filaments on the replication fork DNA. Mutants lacking any or all of these gene products are equally sensitive to DNA damage, although the sensitivity is less than that conferred by a mutation in *recA* [4,50,109,110]. Additionally, *recF*, *recO*, or *recR* mutations also similarly delay induction of LexA-regulated genes following DNA damage [111,112,113]. In *E. coli*, many of the genes upregulated in response to DNA damage are repressed by LexA, which binds to a 20-bp consensus sequence in the operator region of these genes [114,115,116,117]. Once loaded onto single-stranded DNA, RecA becomes conformationally active, serving as a coprotease that promotes the autocatalytic cleavage of the LexA repressor, upregulating transcription from these genes as the cellular concentration of LexA diminishes [118]. Since RecA is required to cleave and inactivate LexA repressors, this supports the idea that there is less activated RecA present at early times after DNA damage in the absence of RecF, RecO, or RecR.

In vitro, interactions between RecO and RecR, and RecF and RecR can be observed in the presence of DNA. RecF, RecO and RecR enhance the ability of RecA to bind and form filaments on single-stranded DNA that is coated with single-strand binding protein (SSB), while limiting growth of the RecA filament to the single-stranded region [95,119,120,121,122], consistent with the general view that these proteins can stabilize RecA filaments in their activated and bound form. Fluorescently labeled fusion proteins of RecF and RecR track with the replisome in live cells [123]. However, RecO is reported to localize separately to loci in the membrane [123], the meaning of which remains unclear. It is interesting to note that *recO* is cotranscribed with an essential G protein, whereas *recR* is cotranscribed with a protein that facilitates integration of membrane-bound G proteins [124]. Speculatively, one could envision RecO providing an anchoring role for the disrupted fork substrate to the membrane where processing may occur.

## 5. RecJ and RecQ Process the Replication Forks Following DNA Damage to Promote Resumption from the Site Where Disruption Occurred

**Figure 5:**
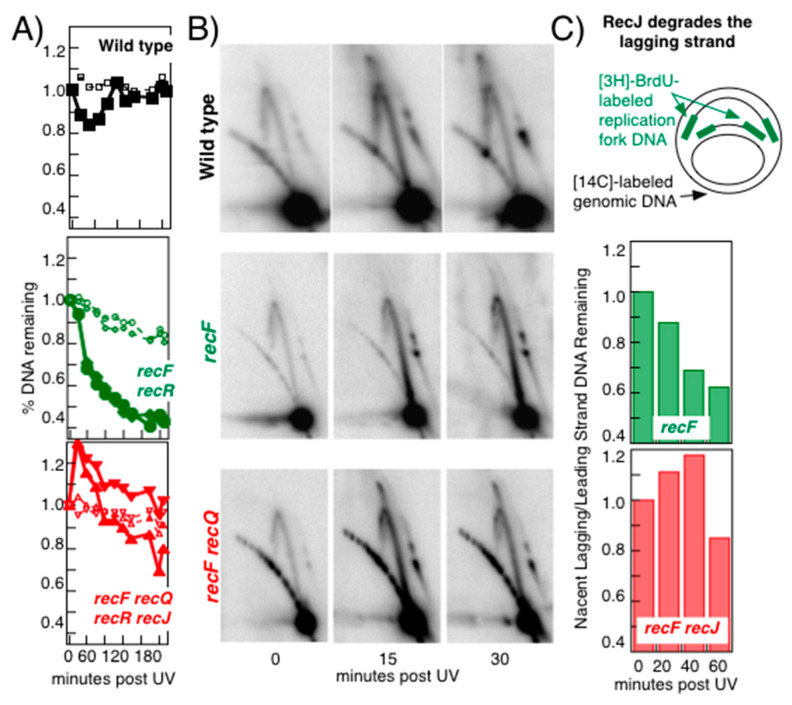
The RecQ helicase and RecJ nuclease degrade the nascent lagging strand of disrupted replication forks. (**A**) *The nascent DNA degradation at disrupted replication forks does not occur in the absence of either RecQ or RecJ*. Degradation of nascent replication fork DNA was measured as described in Figure 4. In *recF* or *recR* mutants, excessive degradation of the nascent DNA occurs after UV. However, this degradation does not occur in the absence of RecJ or RecQ. Filled symbols, nascent DNA; open symbols, total genomic DNA. (**B**) *Inactivation of the RecJ nuclease or RecQ helicase restores the processing intermediates to recF mutants as observed in 2D agarose gels*. Cells containing the plasmid pBR322 were UV irradiated and analyzed by 2D agarose gel analysis as in Figure 4. (**C**) *RecJ preferentially degrades the nascent lagging strand of disrupted replication forks*. Nascent DNA from irradiated cultures was isolated by pulse-labelling cultures with [^3^H]5-bromodeoxyuridine, followed by separation in alkali CsCl gradients. The leading- and lagging-strand nascent DNA remaining at each time point were quantified by using strand-specific probes of the *lacZ* gene. The ratio of the lagging strand signal to the leading strand signal at each time point is plotted. Redrawn from data reported in [51,53].

While RecF, RecO, and RecR function with RecA to maintain replication fork integrity, the processing or degradation that occurs at the replication fork following arrest is mediated by RecQ and RecJ and is necessary for faithful and rapid recovery of replication from the site of disruption [17,53,56,57,63]. RecQ is a 3′–5′ helicase and RecJ is a 5′–3′ single-stranded DNA exonuclease [81,82]. As noted above, replication forks arrested by DNA damage undergo processing and some limited degradation that leads to the 4-way branched intermediates observed in 2D agarose gels. In the absence of RecF, the processing is much more extensive, leading to approximately one half of the nascent DNA being degraded and a loss of the processing intermediate structure seen in irradiated wild-type cells on 2D agarose gels. In the absence of RecJ or RecQ, the nascent DNA processing that normally occurs at disrupted replication forks is not detected [51,53]. Furthermore, inactivation of either RecJ or RecQ prevents the extensive degradation of nascent DNA at the replication fork from occurring in the absence of RecF, RecO, or RecR, and restores the processing intermediates of these mutants as observed by 2D agarose gels (Figure 5A,B) [51,53]. The observations demonstrate that the replication fork intermediates in the cone region are formed through the extrusion of the nascent DNA, and that this is the substrate acted upon by RecJ and RecQ in vivo.

To examine which strands of the nascent DNA are degraded by RecQ and RecJ, the newly synthesized DNA can be pulse labeled with 5-bromodeoxyuridine followed by isolation and enrichment in alkali CsCl gradients. Using probes specific to the leading or lagging strand, it has been shown that the degradation by RecJ and RecQ occurs on the nascent lagging strand (Figure 5C) [51].

**Figure 6:**
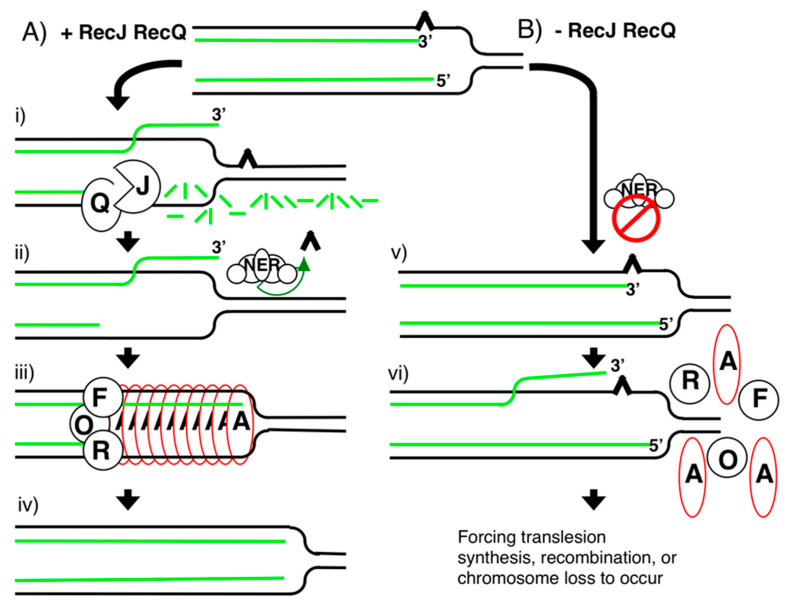
Model for how RecQ and RecJ suppress rearrangements and mutations. (**A**) RecJ-RecQ-mediated degradation of the nascent lagging strand at arrested replication forks (i) restores the lesion-containing region to a double-stranded form that can be repaired (ii) and creates a large substrate (region of single-stranded DNA with homologous duplex DNA) at the fork that RecF-O-R and RecA can bind and stabilize (iii) so that replication can resume (iv). (**B**) In the absence of RecJ-RecQ degradation, the region containing the lesion remains in single-stranded form, preventing its repair, and delaying the recovery until translesion synthesis can occur (v). Additionally, in the absence of RecJ-RecQ processing, RecF-O-R and RecA are unable to bind, leading to recombination at the disrupted site and compromising viability if translesion synthesis cannot occur (vi).

Degradation specific to the nascent lagging strand is consistent with the known polarities and activities of RecJ and RecQ [81,82]. Following the arrest of replication by a template leading-strand lesion, there is expected to be a 3′-end on the nascent leading strand and a 5′-end on the nascent lagging strand (Figure 6). Based on the polarity of RecQ, which loads onto single-stranded DNA and moves in a 3′–5′ direction to displace the complementary strand [82], RecQ would only be able to displace the lagging strand when loaded at the arrested replication fork. Similarly, RecJ, which is a 5′–3′ exonuclease specific for single-stranded DNA [81], would only be able to degrade the lagging strand after it has been displaced by RecQ. Nucleolytic processing of the lagging strand also provides an explanation for why only 50% of the nascent DNA appears to be susceptible to degradation, even when replication is prevented from recovering as is seen in *recF*, *recO*, or *recR* mutants [50,51,53,54].

**Figure 7:**
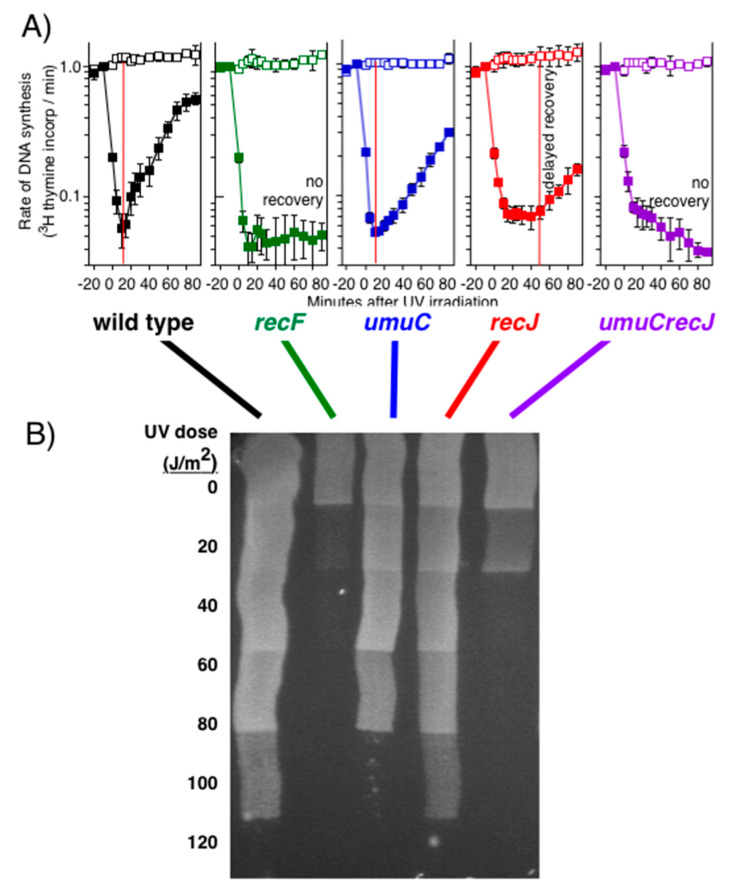
In the absence of RecJ processing, repair cannot occur and replication recovery and cell survival become dependent on translesion synthesis. (**A**) *In the absence of RecJ, the recovery of replication is delayed and becomes entirely dependent on translesion synthesis by PolV, encoded by umuC*. To measure the time at which replication resumes, [^3^H]thymidine was added to cultures for 2 min at the indicated times following either 27 J/m^2^ UV irradiation (filled symbols) or mock irradiation (open symbols) at time 0. The relative amount of DNA synthesis/2 min, ^3^H (squares), is plotted. The time replication resumes is indicated by the red line on each graph. (**B**) *In the absence of RecJ, survival becomes dependent on translesion synthesis by Pol V*. The relative survival of each culture following UV irradiation with the indicated dose is shown. A fresh overnight culture was evenly applied onto a Luria-Bertani medium plate with a cotton swab. The plate was covered, placed under a UV lamp, and the cover was progressively retracted following 20 J/m^2^ exposures. Redrawn from data reported in [55,56].

Although the partial degradation of the nascent lagging strand seems intuitively counterproductive, it serves to move the replication fork back and restore the lesion-containing region to a double-stranded form of DNA that would allow repair to occur. Excision repair requires double-stranded DNA for incision and resynthesis using the complementary strand as a template [99,100]. Consistent with this interpretation, the recovery of DNA synthesis is significantly delayed for 50 min in the absence of RecJ-mediated nascent DNA degradation, and the recovery as well as the survival of the cell become dependent on translesion synthesis by Pol V, arguing that repair enzymes can no longer gain access to the arresting lesion (Figure 7) [56]. The 50 min delay in recovery correlates with the time required for upregulation and activation of Pol V [125,126,127,128], which is the translesion polymerase required for synthesis through UV damage and is responsible for most of the mutagenesis that occurs [129,130]. *umuC* and *umuD*, encode the catalytic and regulatory subunits of the Pol V complex, respectively [131,132]. Both genes are upregulated after UV damage in a LexA-dependent manner. However, activation of translesion synthesis is delayed until UmuD is posttranslationally processed to its active form, termed UmuD’ [133,134,135]. UmuD shares homology with LexA and undergoes a similar autocatalytic cleavage with RecA bound to single-stranded DNA to form UmuD’. The nascent DNA processing would be expected to generate a more extensive three-stranded substrate at the replication fork for RecA to bind and stabilize, thereby ensuring that replication resumes from the same template and same site at which disruption occurred [17,51,63,64,97]. By analogy, RecQ homologs in yeast, Drosophila, and humans have been shown to play critical roles in maintaining processive replication and suppressing the frequency of DNA strand exchanges during replication [136,137,138,139,140,141,142,143,144,145,146].

## 6. Evidence That Lesion Repair Occurs at Disrupted Replication Forks to Prevent Mutations or Rearrangements by Translesion Synthesis or Recombination

**Figure 8:**
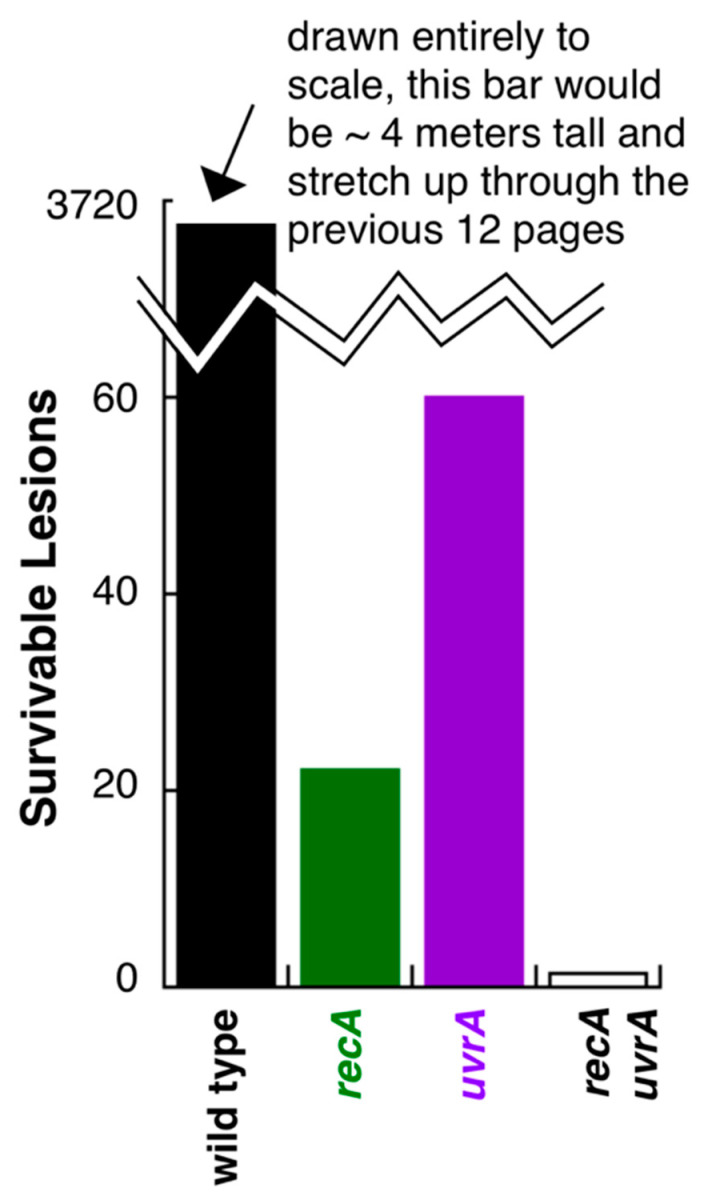
Survival following UV irradiation requires both *rec* and *uvr* function. The number of UV-induced lesions that wild-type, *uvrA*, *recA* and *recA uvrA* cells can survive is plotted. Composed from data reported in [49].

The concept that nucleotide excision repair acts at disrupted replication forks can be inferred from the original survival studies carried out in repair and recombination mutants by Howard-Flanders and Theriot [49]. They found that *E. coli* cultures lacking either recombination (*recA*) or excision repair (*uvrA*) reduced the survival of cultures from doses producing greater than 3500 lesions per genome, to ~20 or 60 lesions per genome, respectively. Further, mutants lacking both *uvrA* and *recA* were similarly sensitive, surviving only 1 or 2 lesions (Figure 8). Fundamental genetics argues that the ability to survive UV-irradiation requires both *uvr* and the *rec* gene products, as survival synergistically increases in the presence of these proteins, and is reduced to nearly zero in their absence. The few lesions survived by these mutants likely represents the number of lesions that nucleotide excision repair can remove before a replisome encounters one in the case of *recA* cells, or that translesion synthesis can bypass in the case of *uvrA* mutants.

Critically, this is not how the authors of this study interpreted these data. The authors’ hypothesis focused on the idea of whether recombination could function as a repair pathway in nonsexual cell cycles, and focused their interpretation exclusively upon the *uvrA* mutant relative to the *uvrA recA* double mutant. In this interpretation, since the *recA* mutation reduced the survival of excision repair mutants from 60 lesions to 2 lesions, they concluded that the difference could represent a function of *recA* promoting survival through a recombinational mechanism that was independent of excision repair. Additionally, while we have immense respect and recognize all that these authors have contributed to this field, in this case, we do not believe the data support this interpretation. In trying to understand the cellular function of a gene product, the mutant’s phenotype should be compared relative to its wild-type counterpart. If the two pathways were operating independently, then survival promoted in the presence of *recA* and *uvrA* might be expected to be additive, or in the range of 100 lesions. Instead, the presence of both these genes allows the parental strain to process and survive several thousand lesions. Indeed, if graphed in its entirety, the bar representing the wild-type strain would extend up twelve pages into the previous article.

The authors’ initial interpretation proved influential as almost every study in the decades of research that followed (and many studies still carried out today) exclusively used nucleotide excision repair mutants to characterize DNA damage-induced recombination and generally interpreted the observed exchanges as repair events [6,7,19,20,21,22,23,24,25,26,27,28,29,30,31,32,33,34,35,36,37,38,39,40,41,42,43,44,45,46,47,48]. The rationale offered for using nucleotide excision repair mutants often was that repair suppressed or masked recombination events making them difficult to observe. In looking at these studies, one cannot avoid noting that cell survival and genomic stability are always severely compromised in the populations where recombination was observed, and lethal doses are typically used in order to generate and observe these strand-exchange events. By analogy, consider the conclusions that would be reached if all human studies on how cells survived DNA damage were conducted exclusively in xeroderma pigmentosum cell lines. Thus, one cannot separate cell death and genomic instabilities from the activity of *rec* gene products functioning in the absence of repair.

One early line of investigation, consistent with the idea that excision repair plays an important role in the RecA-promoted recovery process, comes from the characterization of a phenomenon that was termed “long patch excision repair” [147,148,149]. While characterizing the resynthesis length, or ‘patch size’ of DNA at repair sites following DNA damage, it was observed that the size distribution in UV-irradiated *E. coli* was bimodal. Short patches of 10–12 nucleotides appeared at early times, correlating with normal nucleotide excision repair events. However, at times correlating with when replication recovered, much longer “patches” of DNA synthesis were observed in a process that depended on both *recA* and *uvr* repair genes. Long repair patches localized at DNA replication forks and were made up of fragments that were either >9000 bases or ~1500 bases in length [149]. At the time, the authors concluded that these excision repair-dependent long patches were associated with the efficient recovery of replication and it is notable that their size distribution correlates with those of normal leading and lagging strand synthesis during replication. The results were among the earliest evidence that the recovery of replication depended upon the removal of the blocking lesions by excision repair. A functional synergism between nucleotide excision repair and the RecF pathway was noted by several others in the field who also noted the incongruity of the genetic argument that *rec* and *uvr* pathways operate independently. In each case, the authors found that nucleotide excision repair was a primary component of the *recA*-mediated recovery of replication [150,151,152].

**Figure 9:**
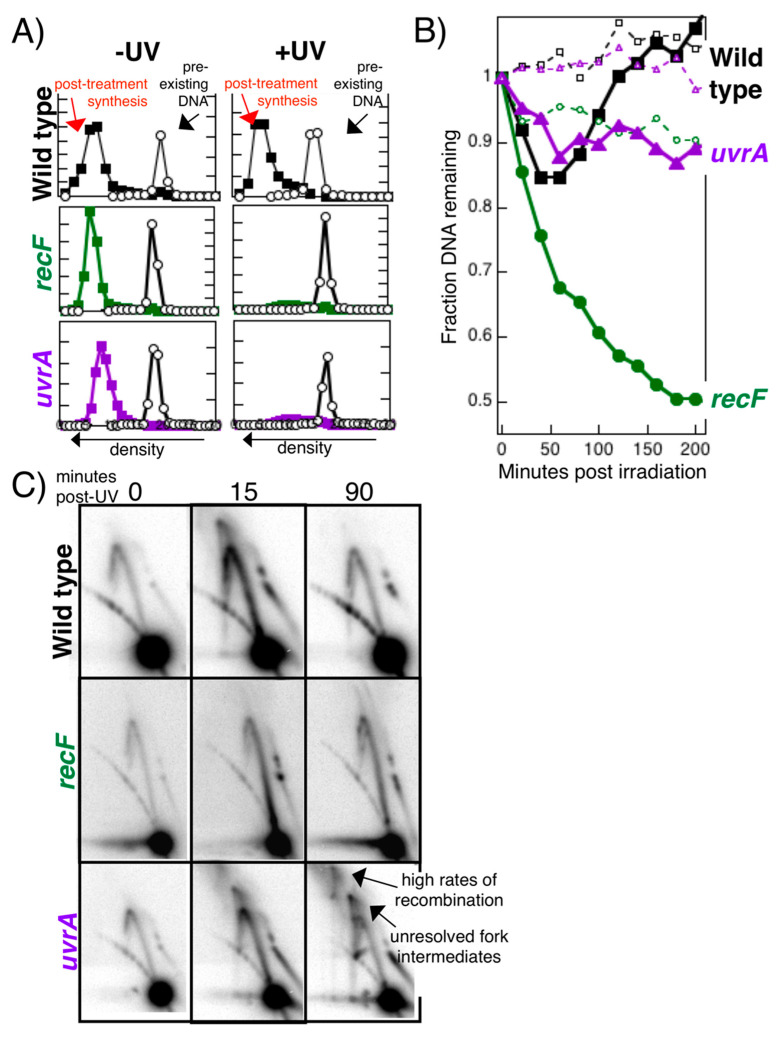
Restoring replication requires both nucleotide excision repair and the RecF pathway. (**A**) *Replication fails to resume in the absence of either RecF or nucleotide excision repair*. The amount of replication occurring within 1 h after UV irradiation with 25 J/m^2^ was analyzed by alkaline CsCl density gradients as described in Figure 4. Circles, ^14^C-prelabeled DNA; squares, ^3^H-replicated DNA. (**B**) *Unlike in recF mutants, the replication fork remains protected in nucleotide excision repair mutants*. The nascent DNA degradation occurring after UV irradiation with 25 J/m^2^ was measured as described in Figure 4. Loss of ^14^C genomic DNA (open symbols) can be compared to the loss of the ^3^H DNA synthesized at the growing fork just prior to irradiation (filled symbols). Squares, wild type; circles, *recF*; triangles, *uvrA*. (**C**) *In the absence of nucleotide excision repair, the intermediates at disrupted replication forks are not resolved and aberrant, higher order recombination intermediates accumulate*. The migration pattern of replicating plasmids in each strain was analyzed by 2D agarose gel analysis as described in Figure 4. Processing intermediates transiently accumulate and then resolve in wild-type cells. No intermediates are observed in *recF* mutants. In *uvrA* mutants, these intermediates persist and go on to form higher-order, recombination intermediates. Redrawn from data reported in [18,53].

The idea that repair occurs at the disrupted replication fork is also supported by assays which directly characterize the DNA damage-arrested replisome. Similar to *recF* mutants, mutants defective in nucleotide excision repair also fail to restore replication after disruption (Figure 9A). The inhibition of replication is dose dependent and mirrors the inhibition seen in the absence of RecF at all doses [18]. However, the phenotype for *uvrA* mutants characterized using nascent DNA processing assays, is distinct from that seen in *recF* mutants (Figure 9B). Whereas, the nascent DNA undergoes extensive degradation in *recF*, it remains protected in the *uvr* mutants and is limited in extent and duration to that seen in wild-type cells. The observation argues that unlike *recF*, the replication fork is maintained and protected in *uvrA* mutants, and the inability to resume is simply due to the unremoved obstructing lesion.

When one examines the structures of the disrupted fork by 2D agarose gel analysis, one observes that replication fork processing begins normally in *uvrA* mutants, but in the absence of lesion removal, these processing intermediates persist and fail to resolve (Figure 9C). In UV-irradiated wild-type cells, these intermediates resolve at a time that correlates with when the lesions are removed [53]. In contrast, the absence of lesion removal in *uvr* mutants leads to higher-order strand-exchange intermediates at later times, consistent with illegitimate recombination events occurring in these dying cells.

**Figure 10:**
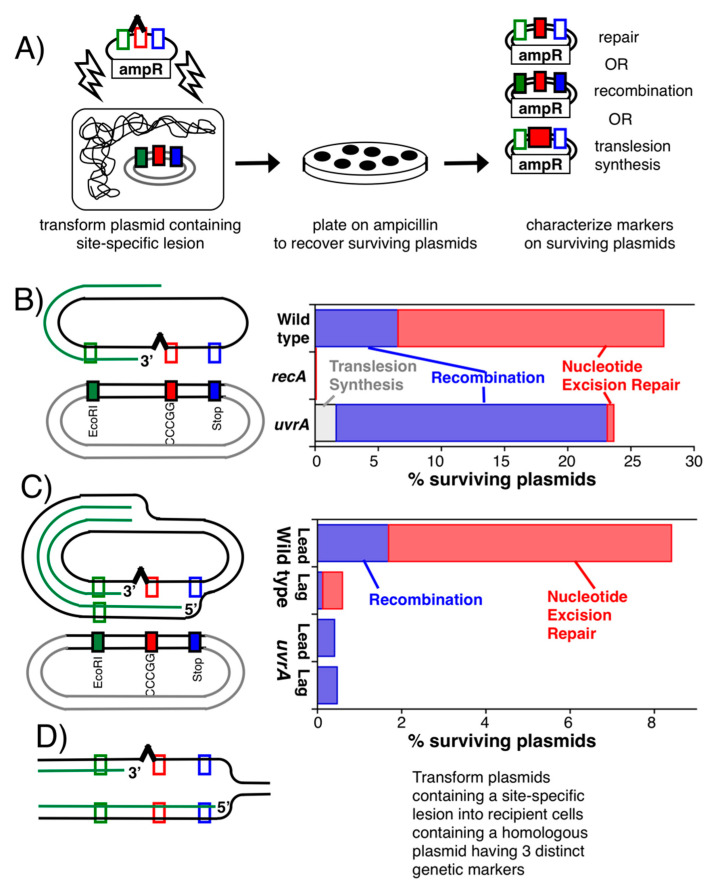
Nucleotide excision repair occurs at disrupted replication forks. (**A**) *Schematic to measure lesion processing on replicating plasmids*. Plasmids containing a site-specific AAF adduct were transformed into cells containing a second endogenous, homologous plasmid with three closely spaced, genetic markers. The markers are designed to allow one to determine whether nucleotide excision repair (surviving plasmids only contained the marker at the lesion site), recombination (surviving plasmids contained multiple genetic markers), or translesion synthesis (contained a base-pair insertion at the middle marker) occurred on the surviving plasmids that are recovered. (**B**) *Nucleotide excision repair events predominate at disrupted replication forks*. When single-stranded plasmids were used in this assay, nucleotide excision repair predominated over recombination events. Few plasmids were recovered in *recA* mutants. In the absence of nucleotide excision repair, survival was reduced and recombination events predominated. (**C**) *Nucleotide excision repair occurs when the lesion is in the leading strand, which disrupts replication, but not the lagging strand, which does not disrupt replication*. The same assay is repeated as in (**B**), except transforming a double-stranded plasmid containing an AAF adduct in either the leading- or lagging-strand template. In wild-type cells, nucleotide excision repair events predominated on the recovered plasmids when the lesion was in the leading strand. When the lesion was in the lagging strand the frequency of repair and plasmids recovered was severely reduced. In *uvrA* mutants, the frequency of plasmids recovered was severely reduced irrespective of whether the lesion was in the leading- or lagging-strand template and recombination events predominated. (**D**) To be scored in this assay, repair events were required to undergo strand exchange and utilize an intermolecular template for repair. *In vivo, the template utilized for repair would be intramolecular, as shown, allowing the reaction to occur with far, far greater frequency and efficiency*. Composed and drawn from data reported in [83,84].

Some of the most direct evidence that lesions are repaired at the disrupted replication fork comes from two elegant studies carried out by Bichara et al. [83,84]. A single-stranded plasmid containing a G-AAF adduct (N-2-acetylaminofluorene attached to the C8 position of guanine), was transformed into cells harboring a second endogenous plasmid that contained three closely spaced genetic markers within a region of homology to the lesion-containing plasmid, surviving plasmids were then recovered and scored for the markers they picked up from the homologous plasmid (Figure 10A). Similar to UV-induced cyclobutane pyrimidine dimers, AAF adducts are bulky lesions that similarly depend upon nucleotide excision repair for removal from the genome [153,154]. The genetic markers were designed to differentiate between nucleotide excision repair (those that only picked up the genetic marker opposite to the lesion on the homologous template), recombination (those that contained extensive regions from the homologous template, having two or all three markers), and translesion synthesis (those in which the middle marker contained an insertion when sequenced). Since nucleotide excision repair cannot occur on single-stranded DNA, all recovered plasmids in this experimental design were forced to undergo strand exchange with the homologous plasmid for either recombination or ‘repair’ to occur. Surprisingly, the majority of events in wild-type cells used nucleotide excision repair rather than recombination to process the plasmid lesion (Figure 10B). Nucleotide excision repair events predominated even though the template containing the lesion was required to occur intermolecularly, using a template supplied by the homologous plasmid. In the absence of nucleotide excision repair, survival was modestly reduced and recombination events predominated. As expected, RecA-mediated strand pairing was required for all these events. The frequency of translesion synthesis was very low, although the lack of SOS induction and Pol IV expression may have hindered this possibility in these single event experiments.

In a critical follow-up study, the authors repeated this assay using double-stranded plasmids that contained the lesion in either the leading or lagging-strand template (Figure 10C). Again because of the genetic markers, only plasmids that utilized the intermolecular template could be scored. The authors observed that on recovered plasmids, nucleotide excision repair events still predominated, particularly when the lesion was found in the leading-strand template. In the absence of nucleotide excision repair, plasmid recovery and survival were significantly reduced, and recombination predominated. When the lesion was in the lagging-strand template, which would not be expected to disrupt replication, few plasmids were recovered.

That nucleotide excision repair was stimulated when the lesion was located in the leading strand, but not the lagging strand, implies that nucleotide excision repair is coupled to replication events. Further and critical to the implications for mechanism, although these experiments were only able to score intermolecular events, the intramolecular template on the lagging strand would clearly be the preferred substrate used following disruption (Figure 10D), which would be further enhanced by the RecJ-mediated degradation of the nascent lagging strand that occurs [51,56]. It would not be unreasonable to expect the intermolecular reaction to occur with an efficiency that is orders of magnitude higher than that of the intermolecular reaction.

Several follow-up studies to this work have placed the lesion on the chromosome [43,44,45,46,47,48]. Unfortunately, these studies reverted to utilizing exclusively repair-deficient mutants, which in our opinion has again removed the biological relevance and led to misinterpretations about what occurs in a wild-type cell that functionally recovers from lesion loads hundreds of times higher than *uvr* mutants. Yet, as supported by their earlier studies, survival in this situation likely occurs through a mechanism that involves both RecF pathway processing at the fork and lesion removal by nucleotide excision repair, so that the replisome can resume.

## 7. Maintaining and Restoring Processive Replication Is Specific to the RecF Pathway

**Figure 11:**
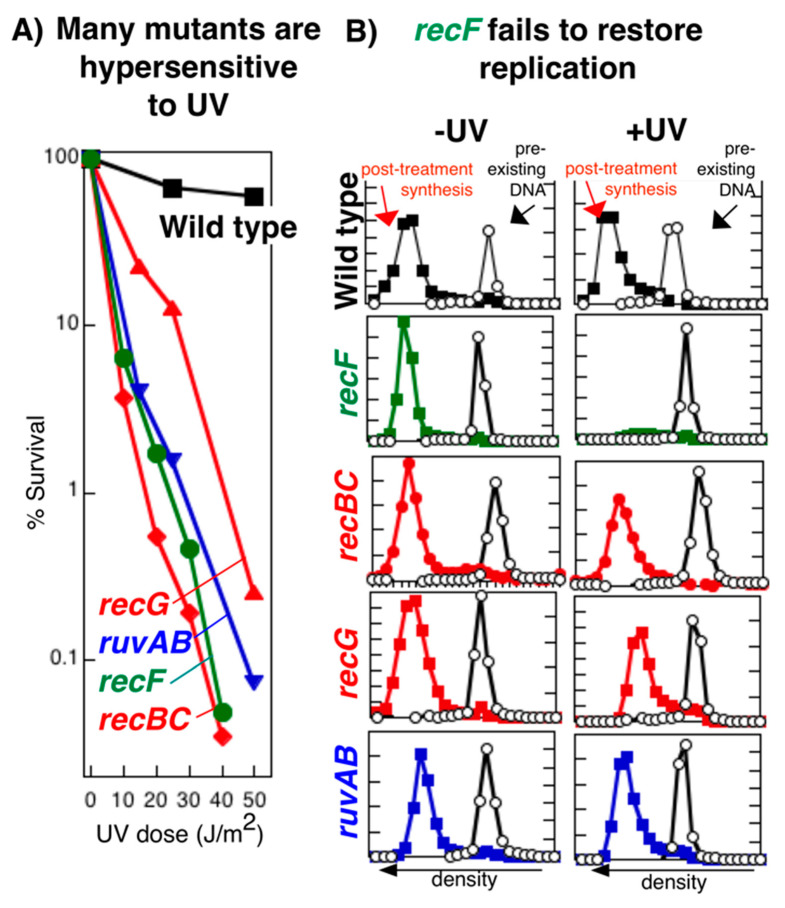
Restoring replication after disruption is specific to the *recF* pathway. (**A**) *recF, recBC, ruvAB,* and *recG* mutants all exhibit high levels of cell lethality following UV-induced DNA damage. The survival of UV-irradiated wild-type, *recF*, *recBC*, *ruvAB*, and *recG* cells at the doses indicated is plotted. (**B**) Yet only *recF* mutants fail to restore replication after UV-induced damage. The amount of DNA synthesized during a 1 h period in UV-irradiated (25 J/m^2^) or mock-irradiated cultures was determined by density labeling the DNA with 5-bromodeoxyuridine and subsequent isolation in isopycnic alkali CsCl gradients. Open circles, DNA synthesized before treatment (^14^C); filled squares, DNA synthesized following treatment (^3^H). The range of ^3^H and ^14^C axes was kept constant for each strain. Redrawn from data presented in [91,164,165].

The processing and restoration of the disrupted replication fork are remarkably specific to the RecF pathway proteins. Similar to *recF* mutants, *recBC*D, *recG* and *ruvAB* mutants are all hypersensitive to DNA damage such as that induced by UV irradiation. As shown in Figure 11A, a moderate dose of 254-nm UV radiation (one from which wild-type cells nearly fully survive) kills more than 99% of cells in all these mutant populations. Based on this hypersensitivity, others have sometimes speculated that these mutants also act at or restore replication forks that encounter DNA damage [87,88,155,156,157,158,159,160,161,162,163]. However, survival assays, by themselves, do not address the cause of death in these mutants. Additionally, none of these studies utilized assays to directly assess whether RecBCD, RecG and RuvAB process the replication fork or affect the ability to restore replication in vivo. In many cases, this led to misconceptions about the cellular role of these other proteins.

Whereas the absence of RecF impairs the recovery of replication after disruption, *recBC*, *recG*, and *ruvAB* mutants each fully recover replication with wild-type kinetics (Figure 11B), indicating that the hypersensitivity of these mutants (i.e., the cause of death) is not associated with a defect in restoring replication after UV damage [50,80,91,107,166]. Although RecBC, RuvAB, and RecG are not essential for replication to resume, it remains possible that these proteins may still act upon or process the disrupted replication forks, and are needed for DNA synthesis to resume at the correct site and using the appropriate template.

**Figure 12:**
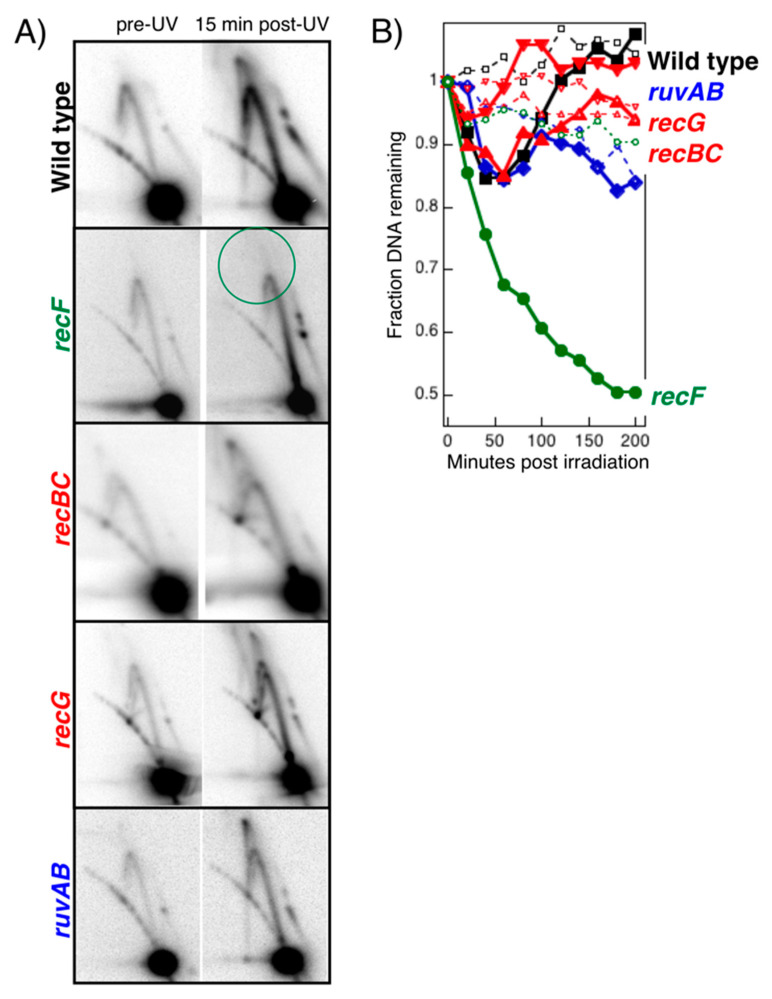
Processing of disrupted replication forks is specific to the RecF pathway. (**A**) The absence of processing intermediates seen at disrupted replication fork is specific to *recF* mutants. DNA from cells containing the plasmid pBR322 were purified, digested with Pvu II, and analyzed by 2D agarose gels immediately before and 15 min after UV irradiation with 50 J/m^2^. Cone region intermediates resembling those in wild-type cells appear in *recBC*, *recG*, and *ruvAB* mutants, but are missing in *recF* mutants. (**B**) The inability to protect the nascent DNA from degradation is specific to the RecF pathway. A 10 s pulse of [^3^H]thymidine is added to [^14^C]thymine-prelabeled cells immediately before the cells are filtered and irradiated with 25 J/m^2^ in nonradioactive medium. The loss (or degradation) of ^14^C genomic DNA (open symbols) can be compared with the loss of the 3H DNA synthesized at the growing fork just before irradiation (filled symbols). Squares, wild type; circles, *recF*; diamonds, *recBC*; triangles, *recG*; inverted triangles, *ruvAB*. Data adapted from [53,80,91].

However, when one examines the replication intermediates in mutants lacking RecBC, RuvAB, or RecG by 2D agarose gels, cone-region intermediates form in each case (Figure 12A) [53,80], indicating that the branch migration activity of RuvAB or RecG, or the helicase-nuclease of RecBCD, is not required to catalyze the fork regression in vivo. Unlike *recF* cells, the nascent DNA degradation in hypersensitive *recBC*, *ruvAB*, and *recG* mutants remains limited and appears similar to wild-type cells following UV-induced DNA damage (Figure 12B) [51,57,91].

These assays strongly argue against the frequently postulated idea that forks collapse to form double-strand breaks at lesions that block replication. This model originally arose from a review article that speculated that if forks collapsed after UV-induced damage, they would form a double-strand break that could account for the UV hypersensitivity of *recBC* mutants [167,168]. Several subsequent studies built upon this hypothesis, reporting that fragmentation could be detected on the chromosome in *recBCD* mutants and that fragmentation was exacerbated following inactivation of the replicative helicase, DnaB [89,90,155,156,157,158,169,170,171,172,173,174,175,176,177]. However, neither this review nor any of the chromosome fragmentation studies employed assays to directly examine the replication fork DNA. On the other hand, when the replication fork DNA was examined directly under these conditions, it was shown that *recBCD* did not act at or contribute to the degradation that occurred at the replication fork in *dnaB* mutants or after UV [51,53,57,58,79]. Further, the fragmentation of chromosomes in *recBCD* mutants has since been shown to localize to the region where replication forks converge to complete replication, but not at sites of replication fork disruption [165,178,179].

One might argue that only a subset or fraction of the arrested replication forks collapse into double-strand breaks. However, *recBC* mutants are as hypersensitive to UV irradiation as *recF* mutants (Figure 11A). Thus, if collapsed replication forks were the cause of *recBC* mutant hypersensitivity, one would predict that there would be a proportionate loss of the arrested Y-shaped structures, and accumulation of linear broken intermediates on 2D agarose gels. Yet no broken linear intermediates are observed at replication forks in UV-irradiated *recBC* cells as analyzed by 2D agarose gels [53,80]. Collapse at disrupted replication forks would also predict that *recBC*D mutants should fail to resume replication after disruption and degrade the nascent DNA at the disrupted fork. However, *recBC* mutants restore replication and process the nascent DNA at the replication fork normally (Figure 11B and Figure 12) [50,107,166]. Taken together, this indicates that replication forks do not collapse or form double-strand breaks, but are maintained in a branched, arrested form, and that RecBCD does not act at damage-arrested replisomes in vivo.

Finally, one could also argue that perhaps replication forks collapse when they encounter other forms of damage, but not when they encounter UV-induced damage. While this certainly could be true, it would not explain why *recBC* mutants are hypersensitive to UV-induced damage. Clearly, some event not associated with the arrested fork arises and requires RecBC function in UV-irradiated cells. If replication fork collapse is not causing death in *recBC* cells after UV, then it is reasonable to consider that it may not necessarily be responsible for causing cell death following other forms of DNA damage.

Based on the biochemical activity of purified proteins and chromosome fragmentation assays, RuvAB and RecG are proposed to promote the regression of replication forks blocked by DNA damage, which is thought to account for the hypersensitivity of *ruvAB* and *recG* mutants [87,89,90,156,159,160,161,172,176,180]. Again however, if true, one would expect that in the absence of either RuvAB or RecG, regressed replication fork intermediates would not accumulate after UV irradiation, and that the recovery of replication would be impaired or delayed. However, as discussed above, the absence of RuvAB or RecG does not prevent the processing that occurs at the replication fork or affect its ability to recover [57,80,91]. The results argue that cell death, in the absence of RecBC, RecG, or RuvAB is caused by a defect that is not directly associated with restoring DNA replication following disruption.

One alternative possibility for the hypersensitivity of these mutants is suggested by recent studies that have shown RecBC and RecG act in the terminus region of the chromosome and play critical roles in completing DNA replication when replication forks converge (for reviews and discussion of the cellular role of these proteins see [165,178,179,181,182,183,184,185]. These studies highlight how although these enzymes were thought to be specific for repairing DNA damage, they are required to process intermediates arising during normal replication. These are roles and functions that really have not been considered or addressed experimentally in mammalian cells, but may restructure how we functionally think about the role and frequency of double-strand breaks in the normal replication cycle.

## 8. Recombination Mediators Are Functionally Conserved between Bacteria and Mammals

In mammalian systems, the role of the BRCA2 pathway and RECQ helicases in maintaining replication forks and suppressing recombination events in mitotic cells is well established [186,187,188,189,190]. Similar to RecF, RecO or RecR mutants, human cells lacking BRCA2 are impaired in their ability to maintain the replication fork and exhibit high levels of genomic instability [186,188]. Loss of BRCA2 leads to more extensive degradation of disrupted replication forks, which recruit Rad51 paralogs that play critical nonrecombinagenic roles in preventing extensive degradation of the nascent DNA at the replication fork and allowing replication to resume [191]. Although homology between RecF, RecO, or RecR is not necessarily apparent at the sequence level, the parallels are strikingly apparent at the phenotypic and functional level [192].

There are five RecQ homologs in humans, RECQ1, BLM, WRN, RECQ4, and RECQ5. Additionally, although each is likely to be temporally or spatially anchored to a unique place in the cell that defines its function, it is notable that all are associated with cancer-prone disease states and genetic instabilities when mutated (reviewed in [193]). Considering that each RecQ homolog is associated with distinct genetic disorders and cancers, it seems likely that they have separate, but likely related, roles in the cell. What defines their unique functions and whether it relates to tissue-specific expression, the nature of the impediment blocking replication, or even differential processing of leading and lagging-strand templates remains unclear at this time. Both RECQ1 and WRN have been reported to protect the nascent DNA at replication forks from degradation under different conditions or in different tissues, similar to their bacterial counterpart [194,195,196]. Similarly, the hallmark of BLM is its ability to suppress exchanges between sister chromatids during replication as shown in Figure 1A [137,197]. Speculatively, this phenotype may suggest a role for this protein in processing and resolving impediments on the lagging-strand template, which are known to allow replication to continue without arresting the progression of the fork [72,73,74,75,76].

Deciphering the mechanisms by which BRCA2 and RECQ operate in mammalian cells, where genome complexity is amplified and the proportion of replicating cells is reduced, can be more challenging. Yet revealing the cellular function of these gene products is key to developing more effective therapies for cancer and enhancing lethality in cells harboring these mutations. The assays available and developed in *E. coli* make a strong argument that we should continue to take advantage of this model organism, especially when clear homologs of bacterial proteins can be identified in mammals.

## Data Availability

Not applicable.

## References

[1] Lederberg J., Tatum E.L. (1953). Sex in Bacteria: Genetic Studies, 1945–1952. Science.

[2] Clark A.J., Margulies A.D. (1965). Isolation and characterization of recombination-deficient mutants of *Escherichia coli* K12. Proc. Natl. Acad. Sci. USA.

[3] Clark A.J. (1967). The beginning of a genetic analysis of recombination proficiency. J. Cell. Physiol..

[4] Horii Z., Clark A.J. (1973). Genetic analysis of the recF pathway to genetic recombination in *Escherichia coli* K12: Isolation and characterization of mutants. J. Mol. Biol..

[5] Howard-Flanders P., Theriot L. (1966). Mutants of *Escherichia coli* K-12 defective in DNA repair and in genetic recombination. Genetics.

[6] Rupp W.D., Howard-Flanders P. (1968). Discontinuities in the DNA synthesized in an Excision-defective strain of *Escherichia coli* following ultraviolet irradiation. J. Mol. Biol..

[7] Rupp W.D., Wilde C.E., Reno D.L., Howard-Flanders P. (1971). Exchanges between DNA strands in ultraviolet-irradiated Escherichia coli. J. Mol. Biol..

[8] Dhar P.K., Devi S., Rao T.R., Kumari U., Joseph A., Kumar M., Nayak S., Shreemati Y., Bhat S., Bhat K.M. (1996). Significance of lymphocytic sister chromatid exchange frequencies in ovarian cancer patients. Cancer Genet. Cytogenet..

[9] Dönmez H., Özkul Y., Uçak R. (1996). Sister chromatid exchange frequency in inhabitants exposed to asbestos in Turkey. Mutat. Res. Mutagen. Relat. Subj..

[10] Murthy M.K., Bhargava M.K., Augustus M. (1997). Sister chromatid exchange studies in oral cancer patients. Indian J. Cancer.

[11] Husain S., Balasubramanian S., Bamezai R. (1992). Sister chromatid exchange frequency in breast cancer cases. Cancer Genet. Cytogenet..

[12] Dhillon V.S., Bhasker R., Kler R.S., Husain S.A. (1995). Sister chromatid exchange (SCE) studies in breast cancer patients: A follow-up study. Cancer Genet. Cytogenet..

[13] Wang L.Y., Lai M.S., Huang S.J., Hsieh C.Y., Hsu M.M., Chen C.J. (1994). Increased sister chromatid exchange frequency in peripheral lymphocytes of nasopharyngeal carcinoma and cervical cancer patients. Anticancer Res..

[14] Dhillon V.S., Kler R.S., Dhillon I.K. (1996). Choromosome instabililty and sister chromatid exchange (SCE) studies in patients with carcinoma of cervix uteri. Cancer Genet. Cytogenet..

[15] Xu X., Wiencke J.K., Niu T., Wang M., Watanabe H., Kelsey K.T., Christiani D.C. (1998). Benzene exposure, glutathione S-transferase theta homozygous deletion, and sister chromatid exchanges. Am. J. Ind. Med..

[16] Strom S.S., Gu Y., Sigurdson A.J., Bailey N.M., Amos C.I., Spitz M.R., Rodriguez M.A., Liang J.C. (1998). Chromosome breaks and sister chromatid exchange as predictors of second cancers in Hodgkin’s disease. Leuk. Lymphoma.

[17] Hanada K., Ukita T., Kohno Y., Saito K., Kato J.-I., Ikeda H. (1997). RecQ DNA helicase is a suppressor of illegitimate recombination in *Escherichia coli*. Proc. Natl. Acad. Sci. USA.

[18] Courcelle J., Crowley D.J., Hanawalt P.C. (1999). Recovery of DNA replication in UV-irradiated *Escherichia coli* requires both excision repair and recF protein function. J. Bacteriol..

[19] Sharma R.C., Sargentini N.J., Smith K.C. (1983). New mutation (mmrA1) in *Escherichia coli* K-12 that affects minimal medium recovery and postreplication repair after UV irradiation. J. Bacteriol..

[20] Smith K.C., Meun D.H. (1970). Repair of radiation-induced damage in *Escherichia coli*: I. Effect of rec mutations on post-replication repair of damage due to ultraviolet radiation. J. Mol. Biol..

[21] Ganesan A.K., Smith K.C. (1971). The duration of recovery and DNA repair in excision deficient derivatives of *Escherichia coli* K-12 after ultraviolet irradiation. Mol. Gen. Genet..

[22] Ganesan A.K. (1974). Persistence of pyrimidine dimers during post-replication repair in ultraviolet light-irradiated *Escherichia coli* K12. J. Mol. Biol..

[23] Youngs D.A., Smith K.C. (1976). Genetic control of multiple pathways of post-replicational repair in uvrB strains of *Escherichia coli* K-12. J. Bacteriol..

[24] Barfknecht T.R., Smith K.C. (1977). Ultraviolet radiation-induced mutability of isogenic uvrA and uvrB strains of *Escherichia coli* K-12 W3110. Photochem. Photobiol..

[25] Sargentini N.J., Smith K.C. (1980). Involvement of genes uvrD and recB in separate mutagenic deoxyribonucleic acid repair path-ways in *Escherichia coli* K-12 uvrB5 and B/r uvrA155. J. Bacteriol..

[26] Wang T.V., Smith K.C. (1981). Effect of recB21, uvrD3, lexA101 and recF143 mutations on ultraviolet radiation sensitivity and genetic recombination in delta uvrB strains of *Escherichia coli* K-12. Mol. Gen. Genet..

[27] Wang T.C., Smith K.C. (1982). Effects of the ssb-1 and ssb-113 mutations on survival and DNA repair in UV-irradiated delta uvrB strains of *Escherichia coli* K-12. J. Bacteriol..

[28] Sharma R.C., Barfknecht T.R., Smith K.C. (1982). Postreplication repair in uvrA and uvrB strains of *escherichia COLI* K-12 IS INHIBITED BY RICH GROWTH MEDIUM. Photochem. Photobiol..

[29] Sargentini N.J., Bockrath R.C., Smith K.C. (1982). Three mechanisms for ultraviolet radiation mutagenesis in *Escherichia coli* K-12 uvrB5: Specificity for the production of back and suppressor mutants. Mutat. Res. Mol. Mech. Mutagen..

[30] Sharma R.C., Smith K.C. (1983). Inducible postreplication repair is responsible for minimal medium recovery in UV-irradiated Escherichia coli K-12. Photochem. Photobiol..

[31] Wang T.C., Smith K.C. (1983). Mechanisms for recF-dependent and recB-dependent pathways of postreplication repair in UV-irradiated *Escherichia coli* uvrB. J. Bacteriol..

[32] Wang T.V., Smith K.C. (1984). recF-dependent and recF recB-independent DNA gapfilling repair processes transfer dimercontaining parental strands to daughter strands in *Escherichia coli* K-12 uvrB. J. Bacteriol..

[33] Wang T.-C.V., Smith K.C. (1985). Role of the umuC gene in postreplication repair in UV-irradiated *Escherichia coli* K-12 uvrB. Mutat. Res. Repair Rep..

[34] Sharma R.C., Smith K.C. (1985). A minor pathway of postreplication repair in *Escherichia coli* is independent of the recB, recC and recF genes. Mutat. Res. Repair Rep..

[35] Sharma R.C., Smith K.C. (1985). A mechanism for rich-medium inhibition of the repair of daughter-strand gaps in the deoxyribonucleic acid of UV-irradiated *Escherichia coli* K12 uvrA. Mutat. Res..

[36] Wang T.C., Smith K.C. (1985). Mechanism of sbcB-suppression of the recBC-deficiency in postreplication repair in UV-irradiated *Escherichia coli* K-12. Mol. Gen. Genet..

[37] Wang T.-C.V., Smith K.C. (1986). Postreplicational formation and repair of DNA double-strand breaks in UV-irradiated *Escherichia coli* uvrB cells. Mutat. Res. Repair Rep..

[38] Sargentini N.J., Smith K.C. (1986). Role of the radB gene in postreplication repair in UV-irradiated *Escherichia coli* uvrB. Mutat. Res. Repair Rep..

[39] Sharma R.C., Smith K.C. (1986). Repair of DNA double-strand breaks in UV-irradiated *Escherichia coli* uvrB recF cells is inhibited by rich growth medium. Mutat. Res. Repair Rep..

[40] Wang T.-C., Smith K.C. (1986). recA (Srf) suppression of recF deficiency in the postreplication repair of UV-irradiated *Escherichia coli* K-12. J. Bacteriol..

[41] Wang T.-C., Smith K. (1988). Different effects of recJ and recN mutations on the postreplication repair of UV-damaged DNA in *Escherichia coli* K-12. J. Bacteriol..

[42] Ganesan A.K., Seawell P.C. (1975). The effect of lexA and recF mutations on post-replication repair and DNA synthesis in Escherichia coli K-12. Mol. Gen. Genet..

[43] Pagès V., Mazón G., Naiman K., Philippin G., Fuchs R.P. (2012). Monitoring bypass of single replication-blocking lesions by damage avoidance in the *Escherichia coli* chromosome. Nucleic Acids Res..

[44] Naiman K., Philippin G., Fuchs R.P., Pages V. (2014). Chronology in lesion tolerance gives priority to genetic variability. Proc. Natl. Acad. Sci. USA.

[45] Laureti L., Demol J., Fuchs R.P., Pagès V. (2015). Bacterial Proliferation: Keep Dividing and Don’t Mind the Gap. PLoS Genet..

[46] Naiman K., Pagès V., Fuchs R.P. (2016). A defect in homologous recombination leads to increased translesion synthesis in E. coli. Nucleic Acids Res..

[47] Laureti L., Lee L., Philippin G., Pagès V. (2017). A non-catalytic role of RecBCD in homology directed gap repair and translesion synthesis. Nucleic Acids Res..

[48] Chrabaszcz É., Laureti L., Pagès V. (2018). DNA lesions proximity modulates damage tolerance pathways in *Escherichia coli*. Nucleic Acids Res..

[49] Howard-Flanders P., Theriot L., Stedeford J.B. (1969). Some Properties of Excision-defective Recombination-deficient Mutants of *Escherichia coli* K-12. J. Bacteriol..

[50] Courcelle J., Carswell-Crumpton C., Hanawalt P.C. (1997). recF and recR are required for the resumption of replication at DNA replication forks in *Escherichia coli*. Proc. Natl. Acad. Sci. USA.

[51] Courcelle J., Hanawalt P.C. (1999). RecQ and RecJ process blocked replication forks prior to the resumption of replication in UV-irradiated *Escherichia coli*. Mol. Gen. Genet..

[52] Courcelle J., Ganesan A.K., Hanawalt P.C. (2001). Therefore, what are recombination proteins there for?. Bioessays.

[53] Courcelle J., Donaldson J.R., Chow K.H., Courcelle C.T. (2003). DNA Damage-Induced Replication Fork Regression and Processing in *Escherichia coli*. Science.

[54] Chow K.-H., Courcelle J. (2004). RecO Acts with RecF and RecR to Protect and Maintain Replication Forks Blocked by UV-induced DNA Damage in *Escherichia coli*. J. Biol. Chem..

[55] Courcelle C.T., Belle J.J., Courcelle J. (2005). Nucleotide Excision Repair or Polymerase V-Mediated Lesion Bypass Can Act To Restore UV-Arrested Replication Forks in *Escherichia coli*. J. Bacteriol..

[56] Courcelle C.T., Chow K.-H., Casey A., Courcelle J. (2006). Nascent DNA processing by RecJ favors lesion repair over translesion synthesis at arrested replication forks in *Escherichia coli*. Proc. Natl. Acad. Sci. USA.

[57] Chow K.H., Courcelle J. (2007). RecBCD and RecJ/RecQ initiate DNA degradation on distinct substrates in UV-irradiated Escherichia coli. Radiat. Res..

[58] Jeiranian H.A., Schalow B.J., Courcelle C.T., Courcelle J. (2013). Fate of the replisome following arrest by UV-induced DNA damage in *Escherichia coli*. Proc. Natl. Acad. Sci. USA.

[59] Wendel B.M., Hollingsworth S., Courcelle C.T., Courcelle J. (2021). UV-induced DNA damage disrupts the coordination between replication initiation, elongation and completion. Genes Cells.

[60] Magner D.B., Blankschien M.D., Lee J.A., Pennington J.M., Lupski J.R., Rosenberg S.M. (2007). RecQ Promotes Toxic Recombination in Cells Lacking Recombination Intermediate-Removal Proteins. Mol. Cell.

[61] Fonville N.C., Blankschien M.D., Magner D.B., Rosenberg S.M. (2010). RecQ-dependent death-by-recombination in cells lacking RecG and UvrD. DNA Repair.

[62] Kusano K., Nakayama K., Nakayama H. (1989). Plasmid-mediated lethality and plasmid multimer formation in an *Escherichia coli* recBC sbcBC mutant. Involvement of RecF recombination pathway genes. J. Mol. Biol..

[63] Hanada K., Iwasaki M., Ihashi S., Ikeda H. (2000). UvrA and UvrB suppress illegitimate recombination: Synergistic action with RecQ helicase. Proc. Natl. Acad. Sci. USA.

[64] Nakayama H., Nakayama K., Nakayama R., Irino N., Nakayama Y., Hanawalt P.C. (1984). Isolation and genetic characterization of a thymineless death-resistant mutant of *Escherichia coli* K12: Identification of a new mutation (recQ1) that blocks the RecF recombination pathway. Mol. Gen. Genet..

[65] Ream L.W., Margossian L., Clark A.J., Hansen F.G., von Meyenburg K. (1980). Genetic and physical mapping of recF in *Escherichia coli* K-12. Mol. Gen. Genet..

[66] Perez-Roger I., Garcia-Sogo M., Navarro-Avino J.P., Lopez-Acedo C., Macian F., Armengod M.E. (1991). Positive and negative regulatory elements in the dnaA-dnaN-recF operon of *Escherichia coli*. Biochimie.

[67] Flower A.M., McHenry C.S. (1991). Transcriptional organization of the *Escherichia coli* dnaX gene. J. Mol. Biol..

[68] Luisi-DeLuca C., Kolodner R. (1994). Purification and characterization of the *Escherichia coli* RecO protein. Renaturation of complementary single-stranded DNA molecules catalyzed by the RecO protein. J. Mol. Biol..

[69] Britton R.A., Powell B.S., Court D.L., Lupski J.R. (1997). Characterization of mutations affecting the *Escherichia coli* essential GTPase era that suppress two temperature-sensitive dnaG alleles. J. Bacteriol..

[70] Britton R.A., Lupski J.R. (1997). Isolation and Characterization of Suppressors of Two *Escherichia coli* dnaG Mutations, dnaG2903 and parB. Genetics.

[71] Britton R.A., Powell B.S., Dasgupta S., Sun Q., Margolin W., Lupski J.R., Court D.L. (1998). Cell cycle arrest in Era GTPase mutants: A potential growth rate-regulated checkpoint in *Escherichia coli*. Mol. Microbiol..

[72] McInerney P., O’donnell M. (2007). Replisome Fate upon Encountering a Leading Strand Block and Clearance from DNA by Recombination Proteins. J. Biol. Chem..

[73] Higuchi K., Katayama T., Iwai S., Hidaka M., Horiuchi T., Maki H. (2003). Fate of DNA replication fork encountering a single DNA lesion during oriC plasmid DNA replication in vitro. Genes Cells.

[74] Pages V., Fuchs R.P. (2003). Uncoupling of Leading- and Lagging-Strand DNA Replication During Lesion Bypass in Vivo. Science.

[75] Svoboda D.L., Vos J.M. (1995). Differential replication of a single, UV-induced lesion in the leading or lagging strand by a human cell extract: Fork uncoupling or gap formation. Proc. Natl. Acad. Sci. USA.

[76] Svoboda D.L., Briley L.P., Vos J.M. (1998). Defective bypass replication of a leading strand cyclobutane thymine dimer in xeroderma pigmentosum variant cell extracts. Cancer Res..

[77] Setlow R.B., Swenson P.A., Carrier W.L. (1963). Thymine dimers and inhibition of DNA synthesis by UL-traviolet irradiation of cells. Science.

[78] Chan G.L., Doetsch P.W., Haseltine W.A. (1985). Cyclobutane pyrimidine dimers and (6-4) photoproducts block polymerization by DNA polymerase I. Biochemistry.

[79] Belle J.J., Casey A., Courcelle C.T., Courcelle J. (2007). Inactivation of the DnaB Helicase Leads to the Collapse and Degradation of the Replication Fork: A Comparison to UV-Induced Arrest. J. Bacteriol..

[80] Donaldson J.R., Courcelle C.T., Courcelle J. (2006). RuvABC is required to resolve holliday junctions that accumulate following replication on damaged templates in *Escherichia coli*. J. Biol. Chem..

[81] Lovett S., Kolodner R.D. (1989). Identification and purification of a single-stranded-DNA-specific exonuclease encoded by the recJ gene of *Escherichia coli*. Proc. Natl. Acad. Sci. USA.

[82] Umezu K., Nakayama K., Nakayama H. (1990). *Escherichia coli* RecQ protein is a DNA helicase. Proc. Natl. Acad. Sci. USA.

[83] Bichara M., Pinet I., Lambert I.B., Fuchs R.P. (2007). RecA-mediated excision repair: A novel mechanism for repairing DNA lesions at sites of arrested DNA synthesis. Mol. Microbiol..

[84] Bichara M., Fuchs R.P., Cordonnier A., Lambert I.B. (2009). Preferential post-replication repair of DNA lesions situated on the leading strand of plasmids in *Escherichia coli*. Mol. Microbiol..

[85] Postow L., Ullsperger C., Keller R.W., Bustamante C., Vologodskii A.V., Cozzarelli N.R. (2001). Positive Torsional Strain Causes the Formation of a Four-way Junction at Replication Forks. J. Biol. Chem..

[86] Fierro-Fernandez M., Hernandez P., Krimer D.B., Schvartzman J.B. (2007). Replication fork reversal occurs spontaneously after digestion but is constrained in supercoiled domains. J. Biol. Chem..

[87] McGlynn P., Lloyd R.G. (2001). Action of RuvAB at Replication Fork Structures. J. Biol. Chem..

[88] McGlynn P., Lloyd R.G., Marians K.J. (2001). Formation of Holliday junctions by regression of nascent DNA in intermediates containing stalled replication forks: RecG stimulates regression even when the DNA is negatively supercoiled. Proc. Natl. Acad. Sci. USA.

[89] Baharoglu Z., Petranovic M., Flores M.-J., Michel B. (2006). RuvAB is essential for replication forks reversal in certain replication mutants. EMBO J..

[90] Le Masson M., Baharoglu Z., Michel B. (2008). RuvA and ruvB mutants specifically impaired for replication fork reversal. Mol. Microbiol..

[91] Donaldson J.R., Courcelle C.T., Courcelle J. (2004). RuvAB and RecG Are Not Essential for the Recovery of DNA Synthesis Following UV-Induced DNA Damage in *Escherichia coli*. Genetics.

[92] Lindsley J.E., Cox M.M. (1990). On RecA protein-mediated homologous alignment of two DNA molecules. Three strands versus four strands. J. Biol. Chem..

[93] Cox M.M., Lehman I.R. (1981). Directionality and polarity in recA protein-promoted branch migration. Proc. Natl. Acad. Sci. USA.

[94] Shan Q., Cox M.M. (1997). RecA Filament Dynamics during DNA Strand Exchange Reactions. J. Biol. Chem..

[95] Shana Q., Bork J.M., Webb B.L., Inman R.B., Cox M.M. (1997). RecA protein filaments: End-dependent dissociation from ssDNA and stabilization by RecO and RecR proteins. J. Mol. Biol..

[96] Cox M.M. (2007). Motoring along with the bacterial RecA protein. Nat. Rev. Mol. Cell Biol..

[97] Hishida T., Han Y.-W., Shibata T., Kubota Y., Ishino Y., Iwasaki H., Shinagawa H. (2004). Role of the *Escherichia coli* RecQ DNA helicase in SOS signaling and genome stabilization at stalled replication forks. Genes Dev..

[98] Setlow R.B., Carrier W.L. (1964). The disappearance of thymine dimers from DNA: An error-correcting mechanism. Proc. Natl. Acad. Sci. USA.

[99] Sancar A., Rupp W. (1983). A novel repair enzyme: UVRABC excision nuclease of *Escherichia coli* cuts a DNA strand on both sides of the damaged region. Cell.

[100] Husain I., Van Houten B., Thomas D.C., Abdel-Monem M., Sancar A. (1985). Effect of DNA polymerase I and DNA helicase II on the turnover rate of UvrABC excision nuclease. Proc. Natl. Acad. Sci. USA.

[101] Caron P.R., Kushner S.R., Grossman L. (1985). Involvement of helicase II (uvrD gene product) and DNA polymerase I in excision mediated by the uvrABC protein complex. Proc. Natl. Acad. Sci. USA.

[102] Van Houten B. (1990). Nucleotide excision repair in *Escherichia coli*. Microbiol. Rev..

[103] Howard-Flanders P., Boyce R.P., Theriot L. (1966). Three loci in *Escherichia coli* K-12 that control the excision of pyrimidine dimers and certain other mutagen products from DNA. Genetics.

[104] Smith C.A., Cooper P.K., Hanawalt P.C., Friedberg E.C., Hanawalt P.C. (1981). DNA Repair A manual of Research ProceduresMeasurement of Repair Repli-Cation by Equilibrium Sedimentation.

[105] Courcelle C.T., Courcelle J. (2006). Monitoring DNA Replication Following UV-Induced Damage in *Escherichia coli*. Methods Enzymol..

[106] Rudolph C.J., Upton A.L., Lloyd R.G. (2008). Maintaining replication fork integrity in UV-irradiated *Escherichia coli* cells. DNA Repair.

[107] Witkin E.M., Roegner-Maniscalco V., Sweasy J.B., McCall J.O. (1987). Recovery from ultraviolet light-induced inhibition of DNA synthesis requires umuDC gene products in recA718 mutant strains but not in recA+ strains of *Escherichia coli*. Proc. Natl. Acad. Sci. USA.

[108] Friedman K.L., Brewer B.J. (1995). Analysis of replication intermediates by two-dimensional agarose gel electrophoresis. Methods Enzymol..

[109] Kolodner R., Fishel R.A., Howard M. (1985). Genetic recombination of bacterial plasmid DNA: Effect of RecF pathway mutations on plasmid recombination in *Escherichia coli*. J. Bacteriol..

[110] Mahdi A.A., Lloyd R.G. (1989). Identification of the recR locus of *Escherichia coli* K-12 and analysis of its role in recombination and DNA repair. Mol. Gen. Genet..

[111] Thoms B., Wackernagel W. (1987). Regulatory role of recF in the SOS response of *Escherichia coli*: Impaired induction of SOS genes by UV irradiation and nalidixic acid in a recF mutant. J. Bacteriol..

[112] Hegde S., Sandler S.J., Clark A.J., Madiraju M.V.V.S. (1995). RecO and recR mutations delay induction of the SOS response in *Escherichia coli*. Mol. Gen. Genet..

[113] Whitby M., Lloyd R.G. (1995). Altered SOS induction associated with mutations in recF, recO and recR. Mol. Gen. Genet..

[114] Brent R., Ptashne M. (1980). The lexA gene product represses its own promoter. Proc. Natl. Acad. Sci. USA.

[115] Brent R., Ptashne M. (1981). Mechanism of action of the lexA gene product. Proc. Natl. Acad. Sci. USA.

[116] Kenyon C.J., Brent R., Ptashne M., Walker G.C. (1982). Regulation of damage-inducible genes in *Escherichia coli*. J. Mol. Biol..

[117] Little J.W., Mount D.W., Yanisch-Perron C.R. (1981). Purified lexA protein is a repressor of the recA and lexA genes. Proc. Natl. Acad. Sci. USA.

[118] Sassanfar M., Roberts J.W. (1990). Nature of the SOS-inducing signal in *Escherichia coli*: The involvement of DNA replication. J. Mol. Biol..

[119] Umezu K., Chi N.W., Kolodner R.D. (1993). Biochemical interaction of the *Escherichia coli* RecF, RecO, and RecR proteins with RecA protein and single-stranded DNA binding protein. Proc. Natl. Acad. Sci. USA.

[120] Umezu K., Kolodner R.D. (1994). Protein interactions in genetic recombination in *Escherichia coli*. Interactions involving RecO and RecR overcome the inhibition of RecA by single-stranded DNA-binding protein. J. Biol. Chem..

[121] Webb B.L., Cox M.M., Inman R.B. (1995). An Interaction between the *Escherichia coli* RecF and RecR Proteins Dependent on ATP and Double-stranded DNA. J. Biol. Chem..

[122] Webb B.L., Cox M.M., Inman R.B. (1997). Recombinational DNA repair: The RecF and RecR proteins limit the extension of RecA filaments beyond single-strand DNA gaps. Cell.

[123] Henrikus S.S., Henry C., Ghodke H., Wood E.A., Mbele N., Saxena R., Basu U., van Oijen A., Cox M.M., Robinson A. (2019). RecFOR epistasis group: RecF and RecO have distinct localizations and functions in *Escherichia coli*. Nucleic Acids Res..

[124] Skretas G., Georgiou G. (2010). Simple Genetic Selection Protocol for Isolation of Overexpressed Genes That Enhance Accumulation of Membrane-Integrated Human G Protein-Coupled Receptors in *Escherichia coli*. Appl. Environ. Microbiol..

[125] Opperman T., Murli S., Smith B.T., Walker G.C. (1999). A model for a umuDC-dependent prokaryotic DNA damage checkpoint. Proc. Natl. Acad. Sci. USA.

[126] Ferentz A.E., Walker G.C., Wagner G. (2001). Converting a DNA damage checkpoint effector (UmuD2C) into a lesion bypass polymerase (UmuD’2C). EMBO J..

[127] Ollivierre J.N., Fang J., Beuning P.J. (2010). The Roles of UmuD in Regulating Mutagenesis. J. Nucleic Acids.

[128] Ollivierre J.N., Sikora J.L., Beuning P.J. (2013). Dimer exchange and cleavage specificity of the DNA damage response protein UmuD. Biochim. Biophys. Acta (BBA)-Proteins Proteom..

[129] Kato T., Shinoura Y. (1977). Isolation and characterization of mutants of *Escherichia coli* deficient in induction of mutations by ultraviolet light. Mol. Gen. Genet..

[130] Bagg A., Kenyon C.J., Walker G.C. (1981). Inducibility of a gene product required for UV and chemical mutagenesis in *Escherichia coli*. Proc. Natl. Acad. Sci. USA.

[131] Reuven N.B., Arad G., Maor-Shoshani A., Livneh Z. (1999). The mutagenesis protein UmuC is a DNA polymerase activated by UmuD’, RecA, and SSB and is specialized for translesion replication. J. Biol. Chem..

[132] Tang M., Shen X., Frank E.G., O’Donnell M., Woodgate R., Goodman M.F. (1999). UmuD’(2)C is an error-prone DNA polymerase, *Escherichia coli* pol V. Proc. Natl. Acad. Sci. USA.

[133] Sommer S., Bailone A., Devoret R. (1993). The appearance of the UmuD’C protein complex in *Escherichia coli* switches repair from homologous recombination to SOS mutagenesis. Mol. Microbiol..

[134] Shinagawa H., Iwasaki H., Kato T., Nakata A. (1988). RecA protein-dependent cleavage of UmuD protein and SOS mutagenesis. Proc. Natl. Acad. Sci. USA.

[135] Nohmi T., Battista J.R., Dodson L.A., Walker G.C. (1988). RecA-mediated cleavage activates UmuD for mutagenesis: Mechanistic relationship between transcriptional derepression and posttranslational activation. Proc. Natl. Acad. Sci. USA.

[136] Davey S., Han C.S., Ramer S.A., Klassen J.C., Jacobson A., Eisenberger A., Hopkins K.M., Lieberman H.B., Freyer G.A. (1998). Fission Yeast rad12 + Regulates Cell Cycle Checkpoint Control and Is Homologous to the Bloom’s Syndrome Disease Gene. Mol. Cell. Biol..

[137] Ellis N.A., Groden J., Ye T.-Z., Straughen J., Lennon D.J., Ciocci S., Proytcheva M., German J. (1995). The Bloom’s syndrome gene product is homologous to RecQ helicases. Cell.

[138] Gray M.D., Shen J.C., Kamath-Loeb A.S., Blank A., Sopher B.L., Martin G.M., Oshima J., Loeb L.A. (1997). The Werner syndrome protein is a DNA helicase. Nat. Genet..

[139] Stewart E., Chapman C.R., Al-Khodairy F., Carr A.M., Enoch T. (1997). rqh1+, a fission yeast gene related to the Bloom’s and Werner’s syndrome genes, is required for reversible S phase arrest. EMBO J..

[140] Yan Z., Xue C., Kumar S., Crickard J.B., Yu Y., Wang W., Pham N., Li Y., Niu H., Sung P. (2019). Rad52 Restrains Resection at DNA Double-Strand Break Ends in Yeast. Mol. Cell.

[141] Kitao S., Shimamoto A., Goto M., Miller R.W., Smithson W.A., Lindor N.M., Furuichi Y. (1999). Mutations in RECQL4 cause a subset of cases of Rothmund-Thomson syndrome. Nat. Genet..

[142] Murray J.M., Lindsay H.D., Munday C.A., Carr A.M. (1997). Role of Schizosaccharomyces pombe RecQ homolog, recombination, and checkpoint genes in UV damage tolerance. Mol. Cell. Biol..

[143] Yamagata K., Kato J., Shimamoto A., Goto M., Furuichi Y., Ikeda H. (1998). Bloom’s and Werner’s syndrome genes suppress hyperrecombination in yeast sgs1 mutant: Implication for genomic instability in human diseases. Proc. Natl. Acad. Sci. USA.

[144] Watt P.M., Hickson I.D., Borts R.H., Louis E.J. (1996). SGS1, a Homologue of the Bloom’s and Werner’s Syndrome Genes, Is Required for Maintenance of Genome Stability in Saccharomyces cerevisiae. Genetics.

[145] Gangloff S., McDonald J.P., Bendixen C., Arthur L., Rothstein R. (1994). The yeast type I topoisomerase Top3 interacts with Sgs1, a DNA helicase homolog: A potential eukaryotic reverse gyrase. Mol. Cell. Biol..

[146] Puranam K.L., Blackshear P.J. (1994). Cloning and characterization of RECQL, a potential human homologue of the *Escherichia coli* DNA helicase RecQ. J. Biol. Chem..

[147] Cooper P.K., Hanawalt P.C. (1972). Heterogeneity of patch size in repair replicated DNA in *Escherichia coli*. J. Mol. Biol..

[148] Cooper P.K., Hanawalt P.C. (1972). Role of DNA Polymerase I and the rec System in Excision-Repair in *Escherichia coli*. Proc. Natl. Acad. Sci. USA.

[149] Cooper P.K. (1982). Characterization of long patch excision repair of DNA in ultraviolet-irradiated *Escherichia coli*: An inducible function under reclex control. Mol. Gen. Genet..

[150] Salaj-Smic E., Petranović D., Petranović M., Trgovcević Z. (1980). Relative roles of uvrA and recA genes in the recovery of Escherichia coli and phage lambda after ultraviolet irradiation. Radiat. Res..

[151] Rudolph C.J., Upton A.L., Lloyd R.G. (2007). Replication fork stalling and cell cycle arrest in UV-irradiated *Escherichia coli*. Genes Dev..

[152] Rothman R.H., Clark A.J. (1977). The dependence of postreplication repair on uvrB in a recF mutant of *Escherichia coli* K-12. Mol. Gen. Genet..

[153] Schmid S.E., Daune M.P., Fuchs R.P. (1982). Repair and mutagenesis of plasmid DNA modified by ultraviolet irradiation or N-acetoxy-N-2-acetylaminofluorene. Proc. Natl. Acad. Sci. USA.

[154] Fuchs R., Seeberg E. (1984). pBR322 plasmid DNA modified with 2-acetylaminofluorene derivatives: Transforming activity and in vitro strand cleavage by the *Escherichia coli* uvrABC endonuclease. EMBO J..

[155] Michel B., Ehrlich S., Uzest M. (1997). DNA double-strand breaks caused by replication arrest. EMBO J..

[156] Seigneur M., Bidnenko V., Ehrlich S., Michel B. (1998). RuvAB Acts at Arrested Replication Forks. Cell.

[157] Michel B. (2000). Replication fork arrest and DNA recombination. Trends Biochem. Sci..

[158] Michel B., Flores M.J., Viguera E., Grompone G., Seigneur M., Bidnenko V. (2001). Rescue of arrested replication forks by homologous recombination. Proc. Natl. Acad. Sci. USA.

[159] McGlynn P., Lloyd R.G. (2001). Rescue of stalled replication forks by RecG: Simultaneous translocation on the leading and lagging strand templates supports an active DNA unwinding model of fork reversal and Holliday junction formation. Proc. Natl. Acad. Sci. USA.

[160] Gregg A.V., McGlynn P., Jaktaji R.P., Lloyd R.G. (2002). Direct Rescue of Stalled DNA Replication Forks via the Combined Action of PriA and RecG Helicase Activities. Mol. Cell.

[161] McGlynn P., Lloyd R.G. (2002). Genome stability and the processing of damaged replication forks by RecG. Trends Genet..

[162] McGlynn P., Lloyd R.G. (2002). Recombinational repair and restart of damaged replication forks. Nat. Rev. Mol. Cell Biol..

[163] Dillingham M.S., Kowalczykowski S.C. (2001). A Step Backward in Advancing DNA Replication: Rescue of Stalled Replication Forks by RecG. Mol. Cell.

[164] Ona K.R., Courcelle C.T., Courcelle J. (2009). Nucleotide excision repair is a predominant mechanism for processing nitrofurazone-induced DNA damage in *Escherichia coli*. J. Bacteriol..

[165] Wendel B., Courcelle C.T., Courcelle J. (2014). Completion of DNA replication in *Escherichia coli*. Proc. Natl. Acad. Sci. USA.

[166] Khidhir M.A., Casaregola S., Holland I.B. (1985). Mechanism of transient inhibition of DNA synthesis in ultraviolet-irradiated E. coli: Inhibition is independent of recA whilst recovery requires RecA protein itself and an additional, inducible SOS function. Mol. Gen. Genet..

[167] Kuzminov A. (1995). Collapse and repair of replication forks in *Escherichia coli*. Mol. Microbiol..

[168] Kuzminov A. (1995). Instability of inhibited replication forks in E. coli. BioEssays.

[169] Michel B., Boubakri H., Baharoglu Z., Lemasson M., Lestini R. (2007). Recombination proteins and rescue of arrested replication forks. DNA Repair.

[170] Flores M.J., Ehrlich S.D., Michel B. (2002). Primosome assembly requirement for replication restart in the *Escherichia coli* holDG10 replication mutant. Mol. Microbiol..

[171] Grompone G., Seigneur M., Ehrlich S.D., Michel B. (2002). Replication fork reversal in DNA polymerase III mutants of *Escherichia coli*: A role for the beta clamp. Mol. Microbiol..

[172] Grompone G., Ehrlich D., Michel B. (2004). Cells defective for replication restart undergo replication fork reversal. EMBO Rep..

[173] Grompone G., Sanchez N., Dusko Ehrlich S., Michel B. (2004). Requirement for RecFOR-mediated recombination in priA mutant. Mol. Microbiol..

[174] Seigneur M., Ehrlich S.D., Michel B. (1999). RecD sbcB sbcD Mutants Are Deficient in Recombinational Repair of UV Lesions by RecBC. J. Bacteriol..

[175] Rothstein R., Michel B., Gangloff S. (2000). Replication fork pausing and recombination or “gimme a break”. Genes Dev..

[176] Seigneur M., Ehrlich S.D., Michel B. (2000). RuvABC-dependent double-strand breaks in dnaBts mutants require recA. Mol. Microbiol..

[177] Uzest M., Ehrlich S.D., Michel B. (1995). Lethality of rep recB and rep recC double mutants of *Escherichia coli*. Mol. Microbiol..

[178] Rudolph C.J., Upton A.L., Stockum A., Nieduszynski C., Lloyd R.G. (2013). Avoiding chromosome pathology when replication forks collide. Nature.

[179] Courcelle J., Wendel B.M., Livingstone D.D., Courcelle C.T. (2015). RecBCD is required to complete chromosomal replication: Implications for double-strand break frequencies and repair mechanisms. DNA Repair.

[180] Flores M.-J., Bierne H., Ehrlich S.D., Michel B. (2001). Impairment of lagging strand synthesis triggers the formation of a RuvABC substrate at replication forks. EMBO J..

[181] Dimude J.U., Midgley-Smith S.L., Stein M., Rudolph C.J. (2016). Replication Termination: Containing Fork Fusion-Mediated Pathologies in *Escherichia coli*. Genes.

[182] Midgley-Smith S.L., Dimude J.U., Rudolph C.J. (2018). A role for 3′ exonucleases at the final stages of chromosome duplication in *Escherichia coli*. Nucleic Acids Res..

[183] Evans J., Maccabee M., Hatahet Z., Courcelle J., Bockrath R., Ide H., Wallace S.S. (1993). Thymine ring saturation and fragmentation products: Lesion bypass, misinsertion and implications for mutagenesis. Mutat. Res. Toxicol..

[184] Wendel B.M., Cole J.M., Courcelle C.T., Courcelle J. (2018). SbcC-SbcD and ExoI process convergent forks to complete chromosome replication. Proc. Natl. Acad. Sci. USA.

[185] Hamilton N.A., Wendel B., Weber E.A., Courcelle C.T., Courcelle J. (2019). RecBCD, SbcCD and ExoI process a substrate created by convergent replisomes to complete DNA replication. Mol. Microbiol..

[186] Schlacher K., Christ N., Siaud N., Egashira A., Wu H., Jasin M. (2011). Double-Strand Break Repair-Independent Role for BRCA2 in Blocking Stalled Replication Fork Degradation by MRE11. Cell.

[187] Siaud N., Barbera M.A., Egashira A., Lam I., Christ N., Schlacher K., Xia B., Jasin M. (2011). Plasticity of BRCA2 function in homologous recombination: Genetic interactions of the PALB2 and DNA binding domains. PLoS Genet..

[188] Schlacher K., Wu H., Jasin M. (2012). A distinct replication fork protection pathway connects Fanconi anemia tumor suppressors to RAD51-BRCA1/2. Cancer Cell.

[189] Lemaçon D., Jackson J., Quinet A., Brickner J.R., Li S., Yazinski S., You Z., Ira G., Zou L., Mosammaparast N. (2017). MRE11 and EXO1 nucleases degrade reversed forks and elicit MUS81-dependent fork rescue in BRCA2-deficient cells. Nat. Commun..

[190] Hambarde S., Tsai C.-L., Pandita R.K., Bacolla A., Maitra A., Charaka V., Hunt C.R., Kumar R., Limbo O., Le Meur R. (2021). EXO5-DNA structure and BLM interactions direct DNA resection critical for ATR-dependent replication restart. Mol. Cell.

[191] Somyajit K., Saxena S., Babu S., Mishra A., Nagaraju G. (2015). Mammalian RAD51 paralogs protect nascent DNA at stalled forks and mediate replication restart. Nucleic Acids Res..

[192] Byrum A.K., Vindigni A., Mosammaparast N. (2019). Defining and Modulating ‘BRCAness’. Trends Cell Biol..

[193] Mojumdar A. (2020). Mutations in conserved functional domains of human RecQ helicases are associated with diseases and cancer: A review. Biophys. Chem..

[194] Zhang J., Lian H., Chen K., Pang Y., Chen M., Huang B., Zhu L., Xu S., Liu M., Zhong C. (2021). RECQ1 Promotes Stress Resistance and DNA Replication Progression Through PARP1 Signaling Pathway in Glioblastoma. Front. Cell Dev. Biol..

[195] Blank A., Bobola M.S., Gold B., Varadarajan S., Kolstoe D.D., Meade E.H., Rabinovitch P.S., Loeb L.A., Silber J.R. (2004). The Werner syndrome protein confers resistance to the DNA lesions N3-methyladenine and O6-methylguanine: Implications for WRN function. DNA Repair.

[196] Datta A., Biswas K., Sommers J.A., Thompson H., Awate S., Nicolae C.M., Thakar T., Moldovan G.-L., Shoemaker R.H., Sharan S.K. (2021). WRN helicase safeguards deprotected replication forks in BRCA2-mutated cancer cells. Nat. Commun..

[197] Chaganti R.S., Schonberg S., German J. (1974). A manyfold increase in sister chromatid exchanges in Bloom’s syndrome lymphocytes. Proc. Natl. Acad. Sci. USA.

